# Stochastic Character Mapping, Bayesian Model Selection, and Biosynthetic Pathways Shed New Light on the Evolution of Habitat Preference in Cyanobacteria

**DOI:** 10.1093/sysbio/syae025

**Published:** 2024-06-27

**Authors:** Giorgio Bianchini, Martin Hagemann, Patricia Sánchez-Baracaldo

**Affiliations:** School of Geographical Sciences, University of Bristol, University Road, Bristol BS81SS, UK; Universität Rostock, Mathematisch-Naturwissenschaftliche Fakultät, Institut für Biowissenschaften, Pflanzenphysiologie, Albert-Einstein-Straße 3, 18059 Rostock, Germany; School of Geographical Sciences, University of Bristol, University Road, Bristol BS81SS, UK

**Keywords:** Bayesian model choice, biosynthetic pathways, compatible solutes, cyanobacteria; habitat preference, stochastic mapping, trait evolution

## Abstract

Cyanobacteria are the only prokaryotes to have evolved oxygenic photosynthesis paving the way for complex life. Studying the evolution and ecological niche of cyanobacteria and their ancestors is crucial for understanding the intricate dynamics of biosphere evolution. These organisms frequently deal with environmental stressors such as salinity and drought, and they employ compatible solutes as a mechanism to cope with these challenges. Compatible solutes are small molecules that help maintain cellular osmotic balance in high-salinity environments, such as marine waters. Their production plays a crucial role in salt tolerance, which, in turn, influences habitat preference. Among the 5 known compatible solutes produced by cyanobacteria (sucrose, trehalose, glucosylglycerol, glucosylglycerate, and glycine betaine), their synthesis varies between individual strains. In this study, we work in a Bayesian stochastic mapping framework, integrating multiple sources of information about compatible solute biosynthesis in order to predict the ancestral habitat preference of Cyanobacteria. Through extensive model selection analyses and statistical tests for correlation, we identify glucosylglycerol and glucosylglycerate as the most significantly correlated with habitat preference, while trehalose exhibits the weakest correlation. Additionally, glucosylglycerol, glucosylglycerate, and glycine betaine show high loss/gain rate ratios, indicating their potential role in adaptability, while sucrose and trehalose are less likely to be lost due to their additional cellular functions. Contrary to previous findings, our analyses predict that the last common ancestor of Cyanobacteria (living at around 3180 Ma) had a 97% probability of a high salinity habitat preference and was likely able to synthesize glucosylglycerol and glucosylglycerate. Nevertheless, cyanobacteria likely colonized low-salinity environments shortly after their origin, with an 89% probability of the first cyanobacterium with low-salinity habitat preference arising prior to the Great Oxygenation Event (2460 Ma). Stochastic mapping analyses provide evidence of cyanobacteria inhabiting early marine habitats, aiding in the interpretation of the geological record. Our age estimate of ~2590 Ma for the divergence of 2 major cyanobacterial clades (Macro- and Microcyanobacteria) suggests that these were likely significant contributors to primary productivity in marine habitats in the lead-up to the Great Oxygenation Event, and thus played a pivotal role in triggering the sudden increase in atmospheric oxygen.

Stochastic mapping is a Bayesian approach in studying trait evolution that can account for uncertainty in the phylogeny of the taxa being analyzed, age estimates of their ancestors, and model parameters (Nielsen 2002; Huelsenbeck et al. 2003). It involves generating many simulations of character evolution according to a continuous-time Markov model, which are constrained to respect observed character states. Uncertainty is accounted for by using different sets of parameters for each simulation, weighted by their posterior probability. In this study, we use stochastic mapping to analyze a complex trait: the evolution of habitat preference in the Cyanobacteria phylum (Garcia-Pichel et al. 2020). We present a methodology for integrating multiple characters in a single analysis, accompanied by a Bayesian model selection approach that makes it possible to reach a conclusion that does not rely on choosing a single model. The use of stochastic mapping allows us to estimate the correlation between different characters using the *D*-test (Huelsenbeck et al. 2003); this approach also enables us to obtain posterior probabilities for all time points along the branches of a phylogenetic tree, rather than just at the internal nodes.

Studies of the evolution of complex traits, consisting of multiple related features, have previously involved the analysis of each feature independently, followed by the comparison of these reconstructions using statistical tests (Felsenstein 1985; Huey and Bennett 1987; Maddison 1990; Huelsenbeck et al. 2003). However, this approach lacks mechanisms to account for correlated evolution in the model itself. In contrast, other methods have made it possible to model multiple characters simultaneously (Pagel 1994; Bianchini and Sánchez‐Baracaldo 2020). This approach offers several advantages, including the ability to account for correlation during all steps of the analysis, estimating how probable the correlation itself is, and increasing the amount of information that can be extracted from the data. While morphological characters are traditionally thought of as “physical” features of an organism, this concept can be extended to encompass any trait that can be categorized into discrete states. In this study, we model the evolution of biochemical pathways by treating an organism’s metabolic capabilities (or its ability to produce specific biomolecules) as morphological characters, allowing us to use a correlated model of evolution (Bianchini and Sánchez‐Baracaldo 2020) to link the history of this trait with the history of the enzymes involved in the pathway.

## Habitat preference in Cyanobacteria

Cyanobacteria are ecologically diverse photoautotrophic bacteria that grow in a wide range of habitats from soil, streams, lakes, oceans, glaciers, deserts, in endolithic communities, and in hot springs <72 °C, and undergo several symbiotic relationships with algae, fungi, plants, and animals (Castenholz et al. 2001). As the only known prokaryotes to have evolved oxygenic photosynthesis, cyanobacterial ancestors played a fundamental role in the oxygenation of Earth’s atmosphere and oceans (Lyons et al. 2014; Schirrmeister et al. 2016). Recent age estimates of the last common ancestor (LCA) of cyanobacteria point to a Mesoarchean origin at ~3200–2800 Ma (Boden et al. 2021; Fournier et al. 2021; Sánchez-Baracaldo et al. 2022) consistent with traces of oxygen in the geological record throughout the Archean eon (4000–2500 Ma; Lyons et al. 2014). While this implies that primary productivity fueled by cyanobacterial ancestors was already established at least 400 Myr before the Great Oxidation Event (GOE) ~2400 Ma, there are still many unknowns when it comes to the biological controls that led to the proliferation (Planavsky et al. 2021) and subsequent dominance (Sánchez-Baracaldo et al. 2022) of cyanobacteria in the Precambrian (i.e., until approximately 540 Ma).

While many geochemistry-based hypotheses explain why it took so long for the oxygenation of the Earth's atmosphere to take place (Lyons et al. 2014; Planavsky et al. 2021), it is less known how cyanobacteria’s origin, subsequent taxonomic diversification, and ecological expansion into different habitats contributed to primary productivity (Schad et al. 2019; Raven and Sánchez-Baracaldo 2021), organic carbon burial over geological time scales (Sánchez-Baracaldo et al. 2014), and the rise of oxygen (Schad et al. 2019; Raven and Sánchez-Baracaldo 2021). Geochemical evidence has shown that atmospheric oxygen (O_2_) levels were low in the Archean, increased to higher levels in the Proterozoic (2500–540 Ma) and reached modern concentration levels across much of the Phanerozoic (540 Ma–present, Lyons et al. 2014). Understanding cyanobacteria’s evolutionary history and the habitat in which they first evolved are fundamental questions when unraveling how their ancestors might have contributed to primary productivity in the Precambrian.

‘Salt tolerance’ is the ability to grow in a hypertonic (e.g., marine, hypersaline) environment, a habitat in which the solution surrounding an organism has a high concentration of solutes (usually inorganic ions) and low water activity compared to its fluids. Cyanobacteria evolved different strategies to achieve salt tolerance, such as employing active ion export mechanisms and synthesizing small organic molecules known as compatible solutes (Brown 1976; Hagemann 2011, 2013). Physiological studies in cyanobacteria have found a correlation between preferred compatible solutes (i.e., sucrose, trehalose, glucosylglycerol, glucosylglycerate, and glycine betaine) and salt tolerance (Mackay et al. 1984; Reed et al. 1986; Hagemann 2011, 2013). For instance, strains accumulating sucrose and/or trehalose can tolerate only sea salt up to 1.05 osmol/kg, while glucosylglycerol and glucosylglycerate enable growth up to 2.10 osmol/kg sea salt, and glycine betaine accumulation allows growth in hypersaline waters up to brines at 4.52 osmol/kg (Reed et al. 1986; Hagemann 2011). Some cyanobacteria can also produce and regulate the biosynthesis of compatible solutes in response to different levels of salinity, enabling them to grow in a range of habitats (Scanlan et al. 2009; Hagemann 2013; Shih et al. 2013). Cyanobacterial biochemical pathways for the synthesis of these compatible solutes have been fully characterized (Supplementary Fig. S1) (Hagemann 2011), and differ in origin (Hagemann 2013) from those found in heterotrophic bacterial groups (Strom and Kaasen 1993; Lunn 2002; Costa et al. 2006; Chen and Murata 2011). These pathways, in most cases, involve 2 gene products, and both genes must be present for the compatible solute to be synthesized (Supplementary Fig. S1, Supplementary Table S1) (Hagemann 2013). It is therefore reasonable to expect that the gain or loss of different genes involved in the same pathway should be correlated.

Habitat preference, defined here as the propensity to growth in a particular habitat (i.e., marine, hypersaline, freshwater), is a complex trait and the by-product of multiple gene products and their interactions. Previous trait evolution studies implementing parsimony (Sánchez-Baracaldo et al. 2005; Dagan et al. 2013; Uyeda et al. 2016; Hammerschmidt et al. 2021), maximum-likelihood (Blank and Sánchez-Baracaldo 2010; Larsson et al. 2011; Latysheva et al. 2012; Uyeda et al. 2016) and stochastic mapping (Sánchez-Baracaldo 2015; Sánchez-Baracaldo et al. 2017b, 2017a) have postulated that the habitat preference of ancestral cyanobacteria was for terrestrial and low salinity environments. A freshwater and terrestrial habitat origin with multiple lineages later radiating into higher salinity environments (Sánchez-Baracaldo et al. 2005; Blank and Sánchez-Baracaldo 2010) has helped to explain the lag between the origin of the LCA of Cyanobacteria and the permanent oxygenation of the atmosphere and the surface (but not deep) ocean during the GOE (Raven and Sánchez-Baracaldo 2021).

Previous stochastic mapping analyses of Cyanobacteria have primarily treated habitat as a ‘morphological’ character in which states were scored based on the type of growth medium and/or isolation habitat (Sánchez-Baracaldo 2015; Sánchez-Baracaldo et al. 2017b), using equal-rates models. However, the implementation of different models (all-rates-different, symmetrical, etc.) led to contrasting results (Nakov et al. 2017; Sánchez-Baracaldo et al. 2017a), highlighting that the choice of model and priors significantly influenced the analysis and results. To address these uncertainties, we have developed a new methodology that simultaneously considers different models (equal-rates, all-rates-different, symmetrical) and multiple sources of information (i.e., the isolation environment and the ability to produce compatible solutes) to study the evolution of habitat preference in Cyanobacteria using a correlated model of evolution. This new methodology allows us to account for uncertainties not previously considered and to put forward new evidence about the ancestral habitat preference of Cyanobacteria in deep time.

## Materials and Methods

### Overview—Stochastic Mapping Analyses and Model Selection

Model selection and parameter estimation are critical steps in phylogenetic analyses (Schultz and Churchill 1999). Ancestral state reconstructions of morphological characters are, in this regard, particularly challenging, as there is often no a priori established model of the process underlying character evolution (Schultz and Churchill 1999; Huelsenbeck and Bollback 2001). However, determining what model to use in the analysis and estimating its parameters is crucial, as different models and different model parameters have previously led to contrasting results (Nakov et al. 2017; Sánchez-Baracaldo et al. 2017a).

Stochastic mapping estimates the posterior probability (PP) of ancestral states (both at the internal nodes of a tree and at every point along a branch) given the observed states for extant taxa (Huelsenbeck et al. 2003). Here, we assigned a high- (*H*) or low-salinity (*L*) habitat preference to each cyanobacterial strain; these data were gathered from the original isolation environment, culture collection data (e.g., growth media), metadata associated with each genome, and experimental studies (Supplementary Table S2).

### Models and Model Parameters

The main parameters for a stochastic mapping analysis are the “transition rates” for the character states, which determine the probability of each kind of state change happening over a certain amount of time. For a binary character, as is habitat preference in this study, there are 2 transition rates that need to be estimated: the H→L rate and the L→H rate. Four different models can be used (listed in Table 1), which differ in the kinds of constraints that are imposed on the rate parameters. In principle, an additional model where both rates are constrained to 0 could be used, but this can be discarded, because it implies that all cyanobacterial strains should have the same habitat preference.

**Table 1.** Transition models for a 2-state character

MODEL	DESCRIPTION	PARAMETERS	RATE MATRIX
Unidirectional (UNI) L→H	Transitions only from L to H	$r_{L\to H}$	$\left(\begin{array}{lll} & *\;r_{L\to H}\; & 0\;*\; \end{array}\right)$
Unidirectional (UNI) H→L	Transitions only from H to L	$r_{H\to L}$	$\left(\begin{array}{lll} & *\;0\; & r_{H\to L}\;*\; \end{array}\right)$
Equal-rates (ER)	Transitions between H and L in both directions, at the same rate	r	$\left(\begin{array}{lll} & *\;r\; & r\;*\; \end{array}\right)$
All-rates-different (ARD)	Transitions between H and L in both directions, at different rates	$r_{L\to H}$ $r_{H\to L}$	$\left(\begin{array}{lll} & *\;r_{L\to H}\; & r_{H\to L}\;*\; \end{array}\right)$

Four possible transition models for a 2-state character are described, highlighting each model’s rate parameters and rate matrix. The values for the diagonal entries in the rate matrix are fully determined by the other matrix entries and are thus omitted (marked with a ∗).

Model selection requires choosing a model out of these options and estimating the rate parameter(s) for the chosen model. A first consideration includes the biological plausibility of each model. In our case, UNI models can be discarded, as there is both evidence for strains living in high-salinity environments arising from groups ancestrally adapted to low-salinity (e.g., marine *Mastigocoleus testarum* and *Nodularia spp.* strains within the mostly freshwater Nostocales; Sukenik et al. 2009; Guida and Garcia-Pichel 2016), as well as freshwater stains arising in ancestrally marine groups (e.g., freshwater *Cyanobium* and *Synechococcus* strains within ancestrally marine picocyanobacteria; Sánchez-Baracaldo et al. 2019).

### Assessing the Effect of Different Parameter Values

**Figure 1.**
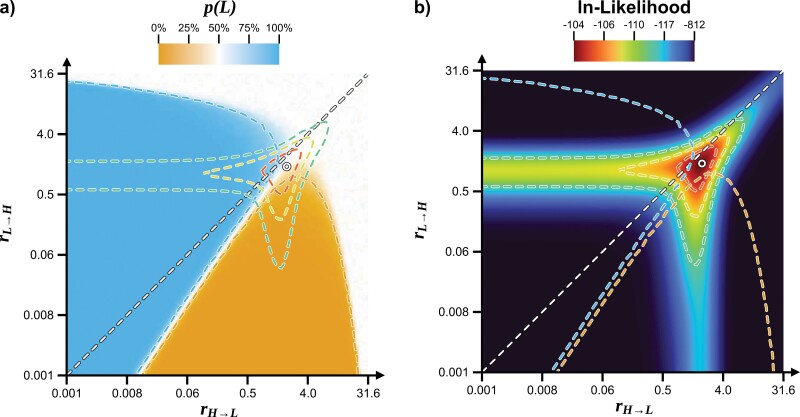
Effect of rate parameters on ancestral state estimates for habitat preference in Cyanobacteria. a) Posterior probability estimates for habitat preference in the last common ancestor (LCA) of Cyanobacteria, in the function of the 2 rate parameters in the model ($r_{H\to L}\;$ and $r_{L\to H}$). With rate parameters in the orange area, a high-salinity ancestral state has a higher probability; with rate parameters in the light blue area, a low-salinity ancestral state has a higher probability. All combinations of rate parameters are allowed under the ARD model, while the ER model only allows values along the diagonal (white dashed line, where $r_{H\to L}=r_{L\to H}$). The white circle highlights the MLE for the rate parameters; the red, yellow, and green dashed lines highlight regions where the likelihood is within 2, 5, or 10 natural log units of the MLE. b) Likelihood of the model in function of the 2 rate parameters. The light blue and orange dashed lines highlight regions where p(L)≥75% and p(H)≥75%, respectively (as in part a). Other markers and dashed lines as in part a. All values reported in this figure were computed using a fixed tree topology and node ages.

Two main models must thus be compared (ER and ARD). To do this, it is beneficial to consider how different combinations of rate parameters can have different effects on habitat preference estimates for the LCA of Cyanobacteria (Fig. 1). Rates in the light blue region indicate that a low-salinity habitat is strongly favored, while rates in the orange region favor high-salinity habitats (Fig. 1a). With rates in the white region, the results of the analysis are uncertain, with about 50% probability for either high- or low-salinity habitat preferences. Under the ARD model, any combination of rates can be used, while under the ER model, only combinations where both rates are equal (highlighted by the dashed white diagonal) are permitted (Fig. 1a).

The most appropriate parameter values to use in the analysis can be determined by looking at the “likelihood landscape” (Fig. 1b), which shows how well each set of parameter values agrees with the data. This makes it possible both to locate the maximum-likelihood estimate for the rates (highlighted for the ARD model by the circle in the figure), and to assess whether the likelihood peak is sharp or broad. In the case of a very sharp peak, the use of a just a single combination of rate parameters can be justified; however, if a broad peak is observed, multiple rate values should be considered, especially if they produce different results.

The MLE for the rate parameters falls within the “uncertain area” (white in Fig. 1a), and the region containing values within 2 natural log units of the MLE overlaps with both the orange (high-salinity) and blue (low-salinity) regions. As a result, an analysis using the MLE value for the rate parameters will not be able to assign the LCA of cyanobacteria to a low- or high-salinity habitat preference state with high probability.

### A Bayesian Approach to Model Selection

Uncertainty in the model parameters can be modeled in a Bayesian approach, by applying a prior distribution over those parameters and thus considering multiple possible values, weighted both by their likelihood and prior probability. If biological information is available, it can be used to inform the priors. Marine-freshwater transitions in microbes are infrequent events (Logares et al. 2009), and this can be implemented as a prior favoring low transition rates (Sánchez-Baracaldo et al. 2017a), such as an exponential distribution with λ=100 (LR, low-rates). In contrast, an empirical Bayes approach can be implemented by determining the location of the prior distribution from the data, by centering the prior around the MLE. A suitable distribution should be chosen, whose density is spread over a reasonable range of values around the MLE. For example, a log-normal distribution with μ=log(MLE) and σ=1 will have about 98% of the prior density between 10% of the MLE and 10 times the MLE, with a median equal to the MLE. Implementation for the ARD and the ER models is possible, respectively by using 2 log-normal distributions centered on the joint MLE for the rates, or by using a single distribution centered on the ER rate MLE.

A Bayesian model averaging approach (Hoeting et al. 1999; Wasserman 2000; Fragoso et al. 2018) can be used to reconcile multiple reconstructions obtained with different models, by considering the posterior probability of each model (P(m | D) in the equation below):


$$\begin{array}{l} P\;\left(H\;|\;D\right)=\underset{m\in M}{\sum }\;P(H,m|D)=\underset{m\in M}{\sum }\;P\;\left(H\;|\;D,m\right)\cdot P\;\left(m\;|\;D\right) \end{array}$$

where H is a hypothesis (such as a low- or high-salinity ancestral habitat preference for cyanobacteria), D is the data, P(H|D) is the posterior probability of the hypothesis given the data, M is the set of all possible models, m is a single model, P(H,m | D) is the joint posterior probability of the hypothesis and model, given the data, and P(H | D,m) is the posterior probability of the hypothesis, given the model and the data.

The posterior probability P(H | D,m) of the hypothesis given the model and data can be computed with a “regular” stochastic mapping analysis, while to compute the posterior probability of the model, a marginal likelihood analysis is needed:


$$\begin{array}{l} P\;\left(m\;|\;D\right)=\;\frac{P\;\left(D\;|\;m\right)\cdot P\;\left(m\right)}{P\;\left(D\right)}=\frac{P\;\left(m\right)}{P\;\left(D\right)}\cdot \underset{\theta \in \Theta }{\int }P\;\left(D\;|\;m,\theta \right)\cdot P(\theta |m) \end{array}$$

where P(m) is the prior probability of the model, P(D | m) is the marginal likelihood of the data given the model, and P(D) is the marginal likelihood of the data. This is a normalization constant that can be obtained without any additional computation as $P\;\left(D\right)=\underset{m\in M}{\sum }\;P\;\left(D\;|\;m\right)\cdot P\;\left(m\right)$.

The marginal likelihood P(D | m) differs from the “simple” likelihood in that it is not conditioned on any particular parameter value:


$$\begin{array}{l} P\;\left(D\;|\;m\right)=\underset{\theta \in \Theta }{\int }P\;\left(D\;|\;m,\theta \right)\cdot P\;(\theta |m) \end{array}$$

where Θ is the (vector) set of all possible parameter values, θ is a parameter vector, P(D | m,θ) is the likelihood of the data, given the model and parameter values, and P(θ|m) is the prior on the parameter values under the specified model. Multiple methods have been developed to compute this integral, such as the harmonic mean estimator (Newton and Raftery 1994), thermodynamic integration (Lartillot and Philippe 2006), stepping-stone sampling (Xie et al. 2011), and more (Fourment et al. 2020); thermodynamic integration and stepping-stone sampling have been shown to produce accurate results (Xie et al. 2011).

Two approaches could be used to select the prior probabilities of the models P(m).   The first is to use a uniform prior (all models are equally probable a priori), while the second consists in determining model prior weights based on how spread out the prior on the parameter values P(θ | m) is for each model. If a higher prior probability is assigned to models with less concentrated priors, this counters the increase in marginal likelihood that results from having a very tight prior around the MLE (Oaks et al. 2019; Klebanov et al. 2021). However, this second approach introduces some new problems, such as how to choose the weighting function and how to compare model spaces with different dimensionalities (Klebanov et al. 2021). Using a non-uniform model prior would be particularly important when comparing parameter priors that are more or less concentrated, but since in our case the priors are similarly spread out (as determined by the σ parameter of the log-normal distribution), we decided to use a uniform prior on the models.

Treating the habitat preference dataset with this empirical Bayes approach estimates a model posterior probability of 0% for the LR model, 55% for the ER model, and 45% for the ARD model. When these are used to weigh the results of the stochastic mapping analyses, they estimate a 67% posterior probability of low salinity habitat preference for the LCA of Cyanobacteria. While this seems to tip the scales in favor of a low salinity ancestral habitat of this group (Sánchez-Baracaldo 2015; Sánchez-Baracaldo et al. 2017b, 2017a), given the lack of confidence (2:1 odds for low salinity habitat preference), more data are needed to study the evolutionary history of habitat preference in Cyanobacteria.

### Analyzing Multiple Characters

**Figure 2.**
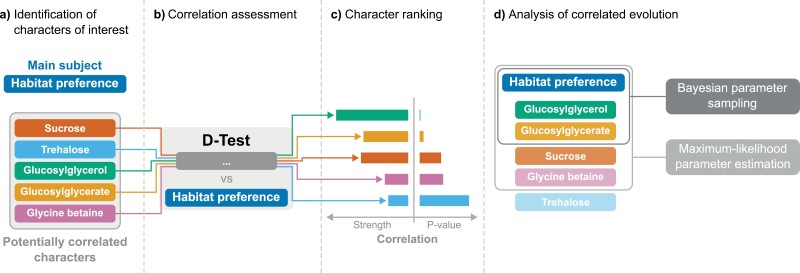
Pipeline for the analysis of the evolution of multiple correlated characters. a) The characters of interest are determined based on experimental evidence, including one character that is the main subject of the investigation (i.e., habitat preference), and others that are expected to be correlated with it. b) The strength and significance of the correlations are determined using a *D*-test. c) The characters are ranked based on the significance of the correlation. d) The characters most significantly correlated with the main subject character are included in an analysis using a correlated model of evolution. When only 2 characters are included, a computationally intensive analysis using Bayesian parameter sampling can be performed. When 4 characters are included, a maximum-likelihood approach to parameter estimation can be implemented to prevent an excessive runtime of the analysis.

When analyzing multiple characters (or even a single character with more than 2 or 3 states), it is unrealistic to perform a detailed analysis (as described above), exploring all possible models and exhaustively sampling the parameter space. Several techniques are, however, available to approximate a thorough approach in a reasonable runtime. After all characters have been identified (Fig. 2a), a *D*-test (Huelsenbeck et al. 2003) can be used to determine the strength, significance, and direction of the correlation between different characters (Fig. 2b). Computation time can then be reduced by identifying and removing characters that show low levels of observed correlation. In fact, the time complexity of exploring the parameter space of a single model is about $O\left(2^{3n}\right)$, where n is the number of characters being considered, while the number of possible models is greater than $2^{2^{2n}}$ (Supplementary Information).

To run a *D*-test, a stochastic mapping analysis is performed separately for each character, including model selection, parameter estimation, and averaging of the results. This approach does not combine multiple characters in the same analysis, and it avoids the exponential runtime increases described above, with a linear O(n) time complexity in the number of characters. Another advantage is that it allows for multiple characters to be analyzed in parallel.

Furthermore, while the number of possible models for each character may be very high, it can be reduced by just considering 3 different models: the equal-rates model (ER, in which all rates are equal), the symmetrical model (SYM, in which rates for symmetrical transitions such as A→B and B→A are the same), and the all-rates-different model (ARD, where each rate can take on a potentially different value). This provides a balanced sampling of the number of rate parameters (respectively, 1, $\frac{s^{2}-s}{2}$, and $s^{2}-s$, where s is the number of character states) without excessively restricting the model space.

Once correlations between the various characters and the phenotype of interest have been estimated, characters can be ranked in order of priority (Fig. 2c) and an appropriate number (e.g., depending on maximum acceptable runtime, number of taxa being analyzed, and available computing resources to perform the analysis, Fig. 2d) can be selected. Finally, the selected characters can be included in a model of correlated evolution (Fig. 2d) (Bianchini and Sánchez‐Baracaldo 2020). For the habitat preference data, we treated habitat preference as being “conditioned” on the other characters. Compared to a “dependent” model (where multiple characters are merged into a single “supercharacter”), this makes it possible to use fewer parameters (about $s^{n}$ instead of $s^{2n}$, where s is the number of states for each character, and n is the number of characters) and to avoid the need for exponentiating large rate matrices.

### Taxon Selection

Amongst prokaryotes, Cyanobacteria are a well-studied bacterial group, partly due to their ecological importance (Díez and Ininbergs 2013), number of genomes available (3706 as of October 2022; Sánchez‐Baracaldo and Cardona 2020; Chen et al. 2021), and their extensive fossil record (Knoll 2012; Schopf 2013; Schirrmeister et al. 2016; Demoulin et al. 2019). Phylogenomic analyses reveal that Cyanobacteria form a monophyletic group within bacteria and their closest relatives are the non-photosynthetic taxa Vampirovibrionia (formerly Melainabacteria) (di Rienzi et al. 2013; Soo et al. 2014) and Sericytochromatia (Soo et al. 2017).

We sampled a broad range of taxonomically diverse cyanobacterial genomes, including many that were not available in previous analyses of habitat evolution in cyanobacteria (Sánchez-Baracaldo 2015; Sánchez-Baracaldo et al. 2017b). Our analyses included a total of 203 bacterial strains, of which 189 were cyanobacteria and 14 represented outgroup taxa: 9 Vampirovibrionia, one Sericytochromatia (di Rienzi et al. 2013; Soo et al. 2014, 2017), and some more distantly related strains in the Terrabacteria group (i.e., 1 Chloroflexi and 3 Firmicutes; Battistuzzi et al. 2004; Battistuzzi and Hedges 2009, Supplementary Table S3). Genome and proteome sequences for these strains were obtained from the GenBank database (Clark et al. 2016); we used BUSCO version 3.0.1 (Simão et al. 2015) with the cyanobacteria_odb9 dataset to assess the completeness of the cyanobacteria genomes (Supplementary Table S4).

### Orthologs, Alignments, and Phylogenetic analyses

Our phylogenomic dataset included 139 protein-coding genes and small subunit (SSU) and large subunit (LSU) rRNA genes and has been updated from previously published studies (Blank and Sánchez-Baracaldo 2010; Sánchez-Baracaldo et al. 2017b). The dataset is available in the Dryad Digital Repository, at https://doi.org/10.5061/dryad.bnzs7h4hq. The included genes are listed in Supplementary Table S5, with a brief description for each gene and the number of strains in which an ortholog for it is found. Orthologs for these genes were identified using BLAST (Camacho et al. 2009) (Supplementary Information).

A maximum-likelihood partitioned phylogenetic analysis was performed using IQ-TREE multicore version 1.6.1 (Nguyen et al. 2015; Kalyaanamoorthy et al. 2017). The best evolutionary model for each gene was determined using IQ-TREE with the -m MFP -te BIONJ options and selecting the model with the lowest BIC score. Each gene was assigned to its own partition, and the previously determined models were used; the partitioned analysis was executed in IQ-TREE using the -sp option, which allows each partition to have a different set of branch lengths, with 1000 ultrafast bootstrap replicates.

The non-cyanobacterial outgroups were excised from the tree using the drop.tip function of the R package ape version 5.3 (Paradis et al. 2004). Between 64 and 100 genes from the dataset were present in each outgroup genome (Supplementary Table S3).

### Fossil Constraints and Molecular Clock Estimation

Stochastic mapping analyses require branch lengths to be expressed in units of time so that rates of change along branches can be estimated (Huelsenbeck et al. 2003; Bianchini and Sánchez‐Baracaldo 2020). A calibrated tree was estimated in Phylobayes version 4.1b (Lartillot and Philippe 2004) with a fixed topology generated by the maximum-likelihood analysis described above, using SSU and LSU ribosomal RNA (4415 total aligned positions) from the 189 cyanobacterial strains. Divergence times were estimated by implementing the uncorrelated gamma multipliers relaxed clock model, with a GTR + CAT model of DNA evolution (Lartillot and Philippe 2004) and a birth-death prior on branch lengths. The root prior was specified as a gamma distribution with a mean 2639 Ma and a standard deviation 179 Myr (which has 95% of the density in the 2300–3000 Ma interval).

Fossil calibrations across Cyanobacteria are well characterized and have been implemented in previous studies (Sánchez-Baracaldo 2015; Sánchez-Baracaldo et al. 2017b, 2019; Boden et al. 2021). In addition to the root calibration mentioned above, we used 5 internal calibration points (Supplementary Table S6), with soft bounds. We ran 2 independent chains and assessed convergence using the tracecomp program from Phylobayes v4.1b. To obtain a tree with the mean branch length estimates, we used the readdiv program in Phylobayes v4.1b; the -v option was used to save 1000 dated trees from the chain to plot age distributions and for stochastic mapping analyses.

### Compatible Solute Biosynthesis Genes

Presence of compatible solute genes (*spsA*, spp, spsA*, *treY, treZ, ggpS, ggpP, ggpS*, *ggpP, gsmT, dmt*) was assessed through BLAST (Camacho et al. 2009) searches using *blastp* version 2.7.1+ (Supplementary Table S7). Query sequences are shown in Supplementary Table S1 and ortholog identification was performed as previously described (Supplementary Information).

We re-aligned ortholog sequences using MAFFT v7.407 (Katoh and Standley 2013) with the --localpair --maxiterate 1000 options. We trimmed the alignments removing all positions with a gap content greater than 85%; we also removed mis-aligned positions at the start and end of each alignment, using the AlignmentViewer online utility (Bianchini 2017).

We used these alignments to build gene trees using MrBayes version 3.2.7a (Ronquist and Huelsenbeck 2003), with a mixed amino acid model prior and among-site rate variation, including a proportion of invariant sites with the remaining site rates drawn from a gamma distribution. Each analysis was run sampling every 1000 generations until the average standard deviation of split frequencies became smaller than 0.01. Chain convergence was assessed with Tracer v1.7.1 (Rambaut et al. 2018).

### Correlation Between Compatible Solutes and Habitat Preference

We used stochastic mapping to determine the amount of correlation between each compatible solute and habitat preference, implementing the *D*-test in sMap v1.0.5 (Bianchini and Sánchez-Baracaldo 2020). To perform the *D*-tests, we first ran a stochastic mapping analysis for each compatible solute independently of the others. For trehalose (Tre), glucosylglycerate (GGA), and glycine betaine (GB), more than 95% of the strains either had a full pathway or a completely missing pathway (only 4 exceptions for Tre, 6 for GGA, and none for GB, Supplementary Table S7); we therefore used binary states, assigning a strain as being able to produce the compatible solute if it had at least one of the genes involved in the pathway. On the other hand, 10 strains had a partial pathway for glucosylglycerol (GG); among them, *Prochlorococcus sp.* MIT 9303 (which has the *ggpP* gene, but lacks *ggpS*) has been experimentally analyzed, and was found to be unable to produce GG (Klähn et al. 2010). Similarly, 13 strains had a partial pathway for sucrose (Suc); these include *Fischerella muscicola* PCC 73103, which has the *spp* gene, but not *spsA* or spsA*, and has also been experimentally verified to be unable to produce Suc (Reed et al. 1984). To account for the possibility of partial GG and Suc pathways, we analyzed all the genes involved in each pathway and determined whether they evolved independently of each other or not. In all cases, we followed an “Empirical Bayes” approach, where the priors on the model parameters are informed by the maximum-likelihood estimates. All stochastic mapping analyses were performed using sMap v1.0.5 (Bianchini and Sánchez-Baracaldo 2020).

For binary characters (Tre, GGA, and GB) we considered the ER and ARD models. We computed maximum-likelihood estimates (MLEs) for the rate parameters under each model, and used these to inform priors for a Bayesian analysis, in which we applied a log-normal prior with μ = log(MLE) and σ=1 (which has about 98% of the density between 10% of the MLE and 10 times the MLE). For parameters with a MLE ≤ 0.01 we used an exponential prior with λ = 100. We performed a stepping-stone analysis (Xie et al. 2011) for each model, determining its marginal likelihood (Supplementary Table S8), and used the marginal likelihood to compute model posterior probabilities (mPPs), assuming an equal prior over the 2 models. Finally, we blended the results of the 2 models using the mPPs as weights.

For GG, we considered the 2 genes involved in the biosynthesis of this compatible solute as either independent of each other, or dependent on each other. For the independent case, we treated each gene as described above, and computed the marginal likelihood of the combined model as the product of the marginal likelihoods of the individual models. For the dependent case, in addition to ER and ARD, we also considered the SYM (symmetrical) model, and treated these 3 models as described above. This resulted in 5 possible models for GG. For Suc we followed a similar approach to GG, considering each gene as independent of the others, the 3 genes as all dependent on each other, and all possible combinations of a single independent gene and 2 dependent genes. This resulted in 14 possible models for Suc.

We used a C# script (Supplementary information) to study the transition rates for the 5 compatible solutes. For Tre, GGA, and GB, we considered a “gain” rate (G), involving state changes from absence (*A*) of the compatible solute to its presence (*P*), and a “loss” rate (L), which corresponds to a transition from *P* to *A*. For GG, we defined the G rate as the sum of the transition rate from *AA* (absence of both genes) to *AP*, *PA*, or *PP*, and the L rate as the sum of transitions from *PP* to *AP*, *PA*, or *AA*. In this case, we also defined an “exchange” rate (E) as the sum of the transition rates between incomplete pathway states (*PA* to *AP* and vice versa). For Suc, the G rate was defined as the sum of all possible transitions from a (putatively) non-functional state to a functional pathway, the L rate as the sum of all possible transitions from a functional to a non-functional pathway, and the E rate as the sum of the remaining transitions (i.e., between functional or non-functional pathways). The C# script was used to plot the posterior distribution of these rates after model averaging and to compute L/G, L/E, and G/E rate ratios (Supplementary Fig. S14).

We then performed a stochastic mapping analysis for the habitat preference character, using the same empirical Bayes approach described above. Finally, we performed *D*-tests by merging the analyses for each compatible solute with the habitat preference analysis.

### Habitat Preference Conditioned on GG and GGA

Based on the results of the *D*-test, we performed a stochastic mapping analysis in which the habitat preference character was conditioned on the presence/absence of GGA and GG. We considered the ARD and ER models for GGA and the all-dependent/ARD model for GG, using the empirical Bayes approach described above to define priors for the rate parameters and implementing a flat Dirichlet prior for conditioned probabilities. GGA was set as an independent character and the 2 genes for GG were set as dependent characters; habitat preference was conditioned on the other 3 characters. We estimated marginal likelihoods using the stepping-stone algorithm (Supplementary Table S9) and used these to compute mPPs and blend the results of the 2 models.

### Habitat Preference Conditioned on Suc, GG, GGA, and GB

We set up 4 sMap analyses including data for Suc, GG, GGA, GB, and habitat preference. GGA and GB were set as independent characters; the 2 genes for GG were set to be dependent on each other, and the 3 genes for Suc were also set to be dependent on each other. Habitat preference was conditioned on the other 7 characters. In each of the 4 analyses, we used a different combination of models for GGA and GB (i.e., ARD for both; ER for both; ARD for GGA and ER for GB; ER for GGA and ARD for GB). In all analyses, we used the all-dependent/ARD model for GG and Suc. In this case, the model was too complex to estimate parameters using a Bayesian approach, therefore we used maximum-likelihood to estimate rates and conditioned probabilities.

Given the complexity of these models, it was not feasible to use the stepping-stone algorithm to estimate marginal likelihoods. However, a comparison between Supplementary Tables S8 and S9 shows that the difference in marginal likelihood between the “composite” models (GGA + GG + Habitat preference) is approximately equal to the difference in marginal likelihood between the individual models (i.e., between the ARD and ER models for GGA). This is because the contribution to the marginal likelihood of the additional characters (GG and Habitat preference) is largely independent of the model implemented for GGA. Therefore, we computed weights for the 4 analyses using the marginal likelihoods of the ARD and ER models for GGA and GB (Supplementary Table S10); these should be approximately equal to the mPPs of the 4 composite models. We also computed AIC and BIC scores (Supplementary Table S10) and used these to compute AIC and BIC weights (Wagenmakers and Farrell 2004). We exported 5000 simulated histories for each of GG, GGA, GB, and habitat with the Merge-sMap utility. Finally, we used a C# script (Supplementary Information) to obtain and plot age estimates for the oldest cyanobacterium with a full pathway for GG, GGA, or GB, and with a high or low salinity habitat preference, as well as for the LCA of crown-group Cyanobacteria, the divergence between Micro- and Macrocyanobacteria, and the LCA of Picocyanobacteria.

Figures depicting phylogenetic trees were created using TreeViewer v2.1.0 and v2.2.0 (Bianchini and Sánchez-Baracaldo 2024). Other figures were created employing Inkscape v1.2.2 (The Inkscape Project 2003) and C# scripts (Supplementary Information), where the Math.NET Numerics library (Math.NET Team 2009) was used to compute statistics, the TreeNode library (Bianchini 2023) was used to manipulate phylogenetic trees, and the VectSharp library (Bianchini 2019) was used for graphics output.

## Results

### Maximum-Likelihood Phylogenomic Analysis

Our maximum-likelihood analysis included 203 bacterial strains (comprising the 189 ingroup cyanobacteria and 14 outgroup taxa, Supplementary Fig. S2, Supplementary Tables S2 and S3). The tree was rooted by using 4 Terrabacteria strains (3 Firmicutes and 1 Chloroflexi) as outgroups. On a broad scale, the tree has recovered well-supported monophyletic groups, which are consistent with previous publications (Sánchez-Baracaldo 2015; Boden et al. 2021; Chen et al. 2021; Fournier et al. 2021; Strunecký et al. 2023). The Vampirovibrionia, a group of heterotrophic soil and gut bacteria, form a monophyletic clade and are sister to the Cyanobacteria (Soo et al. 2014, 2017). *Candidatus* Sericytochromatia bacterium S15B-MN24 CBMW_12 (Sericytochromatia) is sister to both Cyanobacteria and Vampirovibrionia (Soo et al. 2014). The Firmicutes also form a monophyletic group.

Within Cyanobacteria, our analysis confirms the monophyly of previously identified clades (Sánchez-Baracaldo 2015; Boden et al. 2021; Chen et al. 2021; Fournier et al. 2021; Strunecký et al. 2023). *Gloeobacter* strains (*G. violaceus* PCC 7421 and *G. kilaueensis* JS1) form a monophyletic sister group to the other cyanobacteria (Seo and Yokota 2003; Hoffmann et al. 2005; Sánchez-Baracaldo et al. 2005; Blank and Sánchez-Baracaldo 2010; Schirrmeister et al. 2015). As found in other phylogenomic studies (Shih et al. 2013; Sánchez-Baracaldo 2015; Schirrmeister et al. 2015), *Pseudanabaena* strains form a monophyletic group that diverges from other cyanobacteria near the root. *Gloeomargarita* (highlighted in green in Supplementary Fig. S2), the cyanobacterium most closely related to chloroplasts (Ponce-Toledo et al. 2017; Sánchez-Baracaldo et al. 2017b), also diverged early in the evolutionary history of cyanobacteria.

The Micro- and Macrocyanobacteria clades, comprising strains with small (<3 μm) and large (>3 μm) cell diameters, respectively (Sánchez-Baracaldo 2015), are well supported. Within the macrocyanobacteria, the mostly freshwater and filamentous Nostocales form a monophyletic group. *Pleurocapsa* strains—while not all closely related to each other—belong to a well-defined monophyletic clade, as reported in other studies (Sánchez-Baracaldo 2015). Within the Microcyanobacteria, marine and freshwater *Synechococcus* and *Prochlorococcus* strains (which are important primary producers) form a distinct clade of Picocyanobacteria (Scanlan et al. 2009; Sánchez-Baracaldo 2015; Jasser and Callieri 2017; Sánchez-Baracaldo et al. 2019) that are the only cyanobacteria harboring form 1A RubisCo and a special type of carboxysomes (Hess et al. 2001; Badger et al. 2002). As reported elsewhere, many cyanobacterial genera appear to be paraphyletic (Komárek et al. 2014).

Out of 200 nodes with support values assigned to them in the tree, 188 have been recovered with 100% ultrafast bootstrap support. Of the remaining 12 nodes (highlighted in Supplementary Fig. S2), 5 have support values between 81% and 100%, and the other 7 have support values of 61% or lower. The least supported nodes involve internal relationships within the Nostocales (42% support), between Chroococcales and Oscillatoriales (54% support), within Oscillatoriales (61% support), and the position of *Geitlerinema* with respect to *Leptolyngbya* (61% support). All branches leading to major cyanobacterial groups and deep relationships have 100% support.

### Relaxed Molecular Clock Analysis and Evolution of Habitat Preference in Cyanobacteria

Divergence times were estimated in Phylobayes v4.1b (Lartillot and Philippe 2004) using a fixed topology (see analyses above; Supplementary Fig. S2) and the small subunit (SSU) and large subunit (LSU) ribosomal RNA sequences from 189 cyanobacterial taxa. Analyses applied 5 calibration points across cyanobacteria and a gamma distributed root prior with 95% of the density in the 2300–3000 Ma interval. Divergence times were estimated by implementing the uncorrelated gamma multipliers (UGAM) relaxed clock model (Lartillot and Philippe 2004).

**Figure 3.**
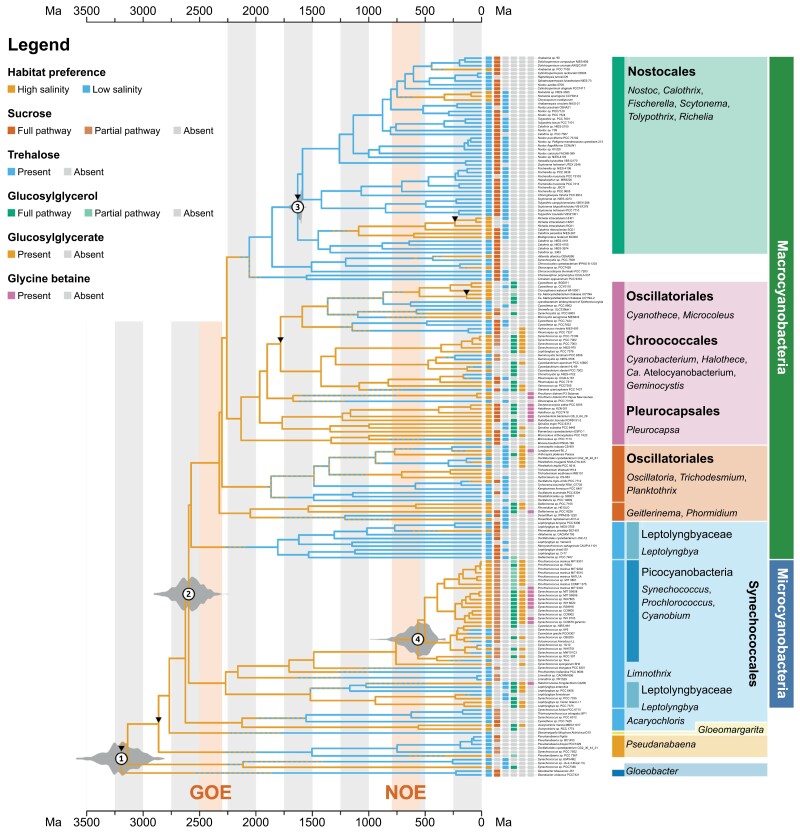
Evolution of habitat preference in Cyanobacteria. Time-calibrated phylogeny of Cyanobacteria showing branch regions with a posterior probability of a high salinity habitat preference greater than 60% (orange), branch regions with a posterior probability of a low salinity habitat preference higher than 60% (blue), and branch regions with a posterior probability of high/low salinity habitat preference between 40% and 60% (dashed pattern alternating orange and blue). Presence or absence of compatible solutes is shown for each strain (see inset legend). Major traditional cyanobacteria groups are also highlighted on the right-hand side; note that many of these are polyphyletic. Posterior distributions are drawn for the age estimates of the LCA of Cyanobacteria (1), the LCA of Micro- and Macrocyanobacteria (2), the LCA of Nostocales (3), and the LCA of Picocyanobacteria (4). Black arrowheads indicate calibration points. GOE: great oxygenation event; NOE: neoproteorozoic oxygenation event.

The results of our molecular clock analyses are broadly similar to previous studies (Sánchez-Baracaldo et al. 2017b, 2019; Boden et al. 2021; Fournier et al. 2021). The LCA of crown group Cyanobacteria has a mean age of 3182 Ma, with an 89% highest posterior density interval (HPD, McElreath 2020) of 2956–3401 Ma and a 95% HPD of 2928–3472 Ma. While consistent with previous estimates (Boden et al. 2021; Fournier et al. 2021), this falls towards the older end of the spectrum, which likely results from the specific taxon selection in this analysis and from the relatively “soft” root age prior (Sánchez-Baracaldo et al. 2022). This node is estimated to have had a preference for high salinity habitats (97%), while multiple lineages of cyanobacteria later evolved a low salinity habitat preference (Fig. 3). These include the early-branching *Gloeobacter* (LCA: 224 Ma, 89% HPD: 79–360 Ma, 95% HPD: 66–470 Ma; low salinity habitat preference PP: 99%) and *Pseudanabaena* lineages (LCA of freshwater strains: 738 Ma, 89% HPD: 422–1070 Ma, 95% HPD: 369–1163 Ma; low salinity habitat preference PP: 98%).

The divergence between Micro- and Macrocyanobacteria is estimated to have happened 2591 Ma (89% HPD: 2407–2758 Ma, 95% HPD: 2391–2820 Ma), and their LCA is estimated to have had a high-salinity habitat preference (PP: 99%). The emergence of these 2 clades was an important evolutionary event in cyanobacteria since they encompass the greatest taxonomic diversity in the phylum (Sánchez-Baracaldo 2015). Picocyanobacteria are a relatively recent group, and their mean age in our tree is 568 Ma (89% CI: 422–732 Ma, 95% CI: 385–770 Ma). This group is also predicted to have had a high salinity habitat preference (PP: 100%). Multiple freshwater lineages appeared within Macro and Microcyanobacteria, such as within the (paraphyletic) Leptolyngbyaceae (LCA: 1807 Ma, 89% HPD: 1378–2322 Ma, 95% HPD: 1279–2374 Ma; low salinity habitat preference PP: 78%), multiple groups within the Oscillatoriales (e.g., *Plantkothrix*, *Oscillatoria*, *Tychonema*, *Cyanothece*), and the Nostocales (low salinity habitat preference PP: 97%). More recent events in which individual strains appear to have independently evolved a low salinity habitat preference include freshwater Picocyanobacteria (e.g., freshwater *Cyanobium* and *Synechococcus*), as well as *Geminocystis*, *Chondrocystis*, *Pleurocapsa sp.* CCALA 161, and *Leptolyngbya foveolarum* (Fig. 3). Our analyses also identified strains with a high salinity habitat preference nested within groups with an ancestrally low salinity habitat preference, especially within the Nostocales: these include marine/brackish Aphanizomenonaceae (LCA: 326 Ma, 89% HPD: 158 – 506 Ma, 95% HPD: 142 – 568 Ma; high salinity habitat preference PP: 91%), the group including *Richelia*, symbiotic *Calothrix*, and *Mastigocoleus* (LCA: 1087 Ma, 89% HPD: 799–1320 Ma, 95% HPD: 743–1375 Ma; high salinity habitat preference PP: 70%), as well as *Anabaena sp.* PCC 7108 (Fig. 3).

Here we focus on 4 nodes (numbered in Fig. 3), illustrating how including different sources of information in the analysis affects the resulting estimates. The nodes are: 1) the LCA of crown-group Cyanobacteria, and 2) the LCA of Micro- and Macrocyanobacteria, both of which represent important evolutionary events for cyanobacteria; and 3) the LCA of the Nostocales and 4) the LCA of Picocyanobacteria, which act as “control” nodes, since there is established evidence for the Nostocales being an ancestrally freshwater group (Sukenik et al. 2009) and for the Picocyanobacteria having originated in a marine environment (Blank and Sánchez-Baracaldo 2010; Sánchez-Baracaldo 2015; Sánchez-Baracaldo et al. 2019).

### Compatible Solute Biosynthesis Genes

We studied the evolution of compatible solute biosynthesis genes by first building a phylogenetic tree (“gene tree”) using the encoded amino acid sequence for each gene. We found a significant number of incongruences between gene trees and the species tree, as well as many internal branches with low support values (Supplementary Figs. S3–S13). While this could be the result of widespread lateral gene transfer events in the history of these genes, a more likely explanation is a lack of phylogenetic signal in the alignments, because of the short sequence length (the average alignment length was around 450 amino acids) combined with the long evolutionary history of cyanobacteria.

We later implemented Stochastic mapping as an alternative sequence-independent approach to address this question, even though the results (Table 2) are still uncertain for some of these genes. Specifically, it is unclear whether the LCA of crown-group Cyanobacteria was able to produce sucrose, as there is a 52% posterior probability that it possessed none of the 3 genes involved in the biosynthesis of this compatible solute, and a 30% probability that it had 2 of them (*spsA* and *spp*). Trehalose shows a similar situation, with a 42% probability of the presence of this compatible solute. For glucosylglycerol (GG), there is a 9% probability that neither *ggpP* nor *ggpS* were present in this ancestor; this means that it is very likely that this ancestor possessed at least one of these genes. However, since both genes are required for the biosynthesis of GG, and there is only a 30% probability of both being present, it is unclear whether the ancestor possessed a functional biosynthetic pathway. Glucosylglycerate (GGA) was most likely present in this ancestor (93%). Finally, it is unclear whether it could produce glycine betaine (GB), but it is more likely that it could not (23% probability of presence).

**Table 2.** Presence or absence of compatible solutes in cyanobacterial ancestors

	Compatible solutes (%)											
	Suc			Tre		GG			GGA		GB	
Node	P	I	A	P	A	P	I	A	P	A	P	A
LCA of cyanobacteria	44	4	52	42	58	30	61	9	**93**	7	23	77
LCA of micro- and macrocyanobacteria	28	4	68	40	60	57	38	5	**94**	6	18	**82**
LCA of nostocales	**90**	6	4	**81**	19	13	1	**85**	17	**83**	5	**95**
LCA of picocyanobacteria	**94**	0	6	8	**92**	64	21	15	**99**	1	16	**84**

Summary of stochastic mapping analyses in which each compatible solute was analyzed independently. LCA: last common ancestor. P: presence of a complete pathway for the compatible solute (for Suc, this includes all states where *spsA* is present or *spp* is present with spsA*, see Discussion; for other compatible solutes this includes the state where both genes are present). I: presence of an incomplete pathway for the compatible solute. A: absence of all genes for the compatible solute. Values are posterior probabilities reported as percentages; values higher than 80% are highlighted in bold.

The presence of Suc and Tre in the LCA of Macro- and Microcyanobacteria is also unclear (68% probability of absence of all genes for Suc, 40% presence of Tre). In contrast, it is more likely that the LCA of Macro- and Microcyanobacteria had the full GG pathway (57% presence of both genes, 5% absence of both genes), though there is still a significant probability of it possessing only one gene (38%). GGA was also probably present in this ancestor (94%), while GB was absent (18%). The LCA of the Nostocales could likely produce Suc and Tre (72% presence of spsA* and *spp*, 18% presence of all 3 genes for Suc; 81% presence of Tre). However, it probably could not produce any other compatible solute (85% absence of both genes for GG, 17% presence of GGA, 5% presence of GB). The LCA of the Picocyanobacteria has a 94% probability of possessing the *spsA* gene for Suc, while the other 2 genes were likely absent (< 4%). Tre was also likely absent in this ancestor (8% probability of presence) and GG was probably present (85% presence of at least one gene in the pathway). GGA was present (99%), and GB was probably absent (16%).

### Transition Rates

Further information about the processes underlying the characters we analyzed can be gained by examining the parameters of the evolutionary model fitted to the observed data (Supplementary Fig. S14). For the compatible solute genes, we can identify 3 kinds of events: gene loss (L, e.g., a non-sense mutation, a frameshift, deletion Mira et al. 2001; Kuo and Ochman 2009); gene gain (G, e.g., horizontal gene transfer, de novo gene origin, Tautz and Domazet-Lošo 2011), and gene exchange (E, i.e., simultaneous gene losses and gains). The models we used for GGA, GB, and Tre only involve L and G events. After model averaging, the median L/G rate ratios for GGA, GB, and Tre are respectively 287, 9, and 1. This indicates that for GGA and GB, loss events are more likely than gain events, while for Tre the 2 events happen with the same frequency. For GG and Suc, the all-dependent/ARD model allows L, G, and E events. For GG, the median L/G rate ratio is 19, while L/E and G/E are 131 and 6.8, respectively. This highlights, again, that losses are much more likely than gains, and that exchanges are the rarest kind of event. In the case of Suc, the L/G rate ratio is 0.66, while L/E and G/E are 1.8 and 2.7, respectively. Losses of Suc are thus (slightly) less likely than gains, and exchange events are rarer than losses and gains.

### Correlation Between Compatible Solutes and Habitat Preference

The correlation between compatible solute biosynthesis genes can be assessed from the results of our D-test analyses. This statistical test is described by Huelsenbeck et al. (2003), and we provide additional details in the Supplementary Information. A summary of the meaning of D-test values is shown in Table 3. In brief, high values for the *D* and *d*_*i,j*_ statistics indicate a strong correlation, while low values for the posterior-predictive *P* and *P_i,j_* statistics indicate a significant correlation (compared to the null hypothesis of the 2 characters evolving independently).

**Table 3.** Interpretation of the results of a *D*-test

Statistic	Values	Interpretation
*D*	> 0% or <0%	Strong positive or negative correlation between the 2 characters.
	Close to 0%	Weak correlation between the 2 characters.
*d*_*i,j*_	> 0% or <0%	Strong positive or negative correlation between states *i* and *j*.
	Close to 0%	Weak correlation between states *i* and *j*.
*P*	Close to 0%	Significant correlation between the 2 characters.
	> 0%	Non-significant correlation between the 2 characters.
*P*_*i,j*_	Close to 0%	Significant correlation between states *i* and *j*.
	> 0%	Non-significant correlation between states *i* and *j*.

The results of a *D*-test include values for 4 main statistics. The *D* and *d*_*i,j*_ statistics provide an indication of the strength of the correlation, while the posterior predictive *P* and *P*_*i,j*_ values provide an indication of the statistical significance of the correlation. The range within which values can be considered “close” or “far” from 0% must be chosen by individual researchers; we consider 5% to be a reasonable threshold.

Our *D*-test analyses (Table 4) reveal that GG (posterior predictive *P* = 0.003) is the compatible solute most significantly associated with habitat preference. The absence of both genes for GG is strongly correlated with a low salinity habitat preference, while the presence of both genes is associated with a high salinity habitat preference. Character states where only one gene for GG is present are also associated with a high salinity habitat preference, though not as strongly. GGA is also significantly associated with habitat preference (*P* = 0.0155). The absence of GGA is associated with a low salinity habitat preference, while its presence is associated with a high salinity habitat preference.

**Table 4.** *D*-test for correlation between compatible solutes and habitat preference

	Comparison	*D* / *d*_*i,j*_	*P* / *P*_*i,j*_
Sucrose	Overall	19%	8.65%
	All absent vs. H	2.6%	4.8%
	All present vs. H	−1.5%	9.0%
Trehalose	Overall	8.0%	19.85%
	Absent *vs* H	1.9%	22%
Glucosylglycerol	Overall	29%	0.3%
	All absent vs. H	−7.1%	0.3%
	All present vs. H	5.9%	0.3%
Glucosylglycerate	Overall	18%	1.55%
	Absent vs. H	−4.4%	1.6%
Glycine betaine	Overall	9.4%	9.45%
	Absent vs. H	−2.3%	9.5%

The rows marked “overall” contain the overall *D* value and posterior predictive *P* value for the compatible solute. The other rows contain *d*_*i,j*_ and *P*_*i,j*_ values for specific combinations of states for the compatible solute and habitat preference (*H* = high salinity habitat preference). Negative values of *D*/ *d*_*i,j*_ indicate a negative correlation, while positive values indicate positive correlation. For example, absence of GGA is negatively correlated with high salinity habitat preference. For 2-state characters (i.e., Tre, GGA, and GB), *P*_*i,j*_ values are identical for all combination of states, and *d*_*i,j*_ values are also identical, up to a change in sign; hence, only one value is reported (e.g., the *d*_*i,j*_ between presence of Tre and high salinity habitat preference is −1.9%, with a *d*_*i,j*_ of 22%; the same values apply to the comparison between absence of Tre and low salinity habitat preference). For Suc and GG, *P*_*i,j*_ values for comparisons against the low salinity habitat preference state are identical to the ones against the high salinity habitat preference, while *d*_*i,j*_ values have the same magnitude but opposite sign (e.g., the *d*_*i,j*_ between the absence of all genes for Suc and low salinity habitat preference is −2.6%, with a *P*_*i,j*_ of 4.8%). For these compatible solutes, state combinations with a *P*_*i,j*_ higher than 10% are not reported.

The association between GB and habitat preference is less significant than the previous 2 compatible solutes (*P* = 0.0945); nevertheless, the presence of GB is associated with high salinity habitat preference and absence is associated with low salinity habitat preference. The association between Suc and habitat preference has similar significance (*P *= 0.0865); here, the strongest association is between the absence of all 3 genes involved in Suc biosynthesis and high salinity habitat preference. Finally, statistical support for the association between Tre and habitat preference is very weak (*P* = 0.1985); the absence of Tre is associated with high salinity habitat preference, while its presence is associated with low salinity habitat preference.

### Stochastic Mapping Analyses

**Figure 4.**
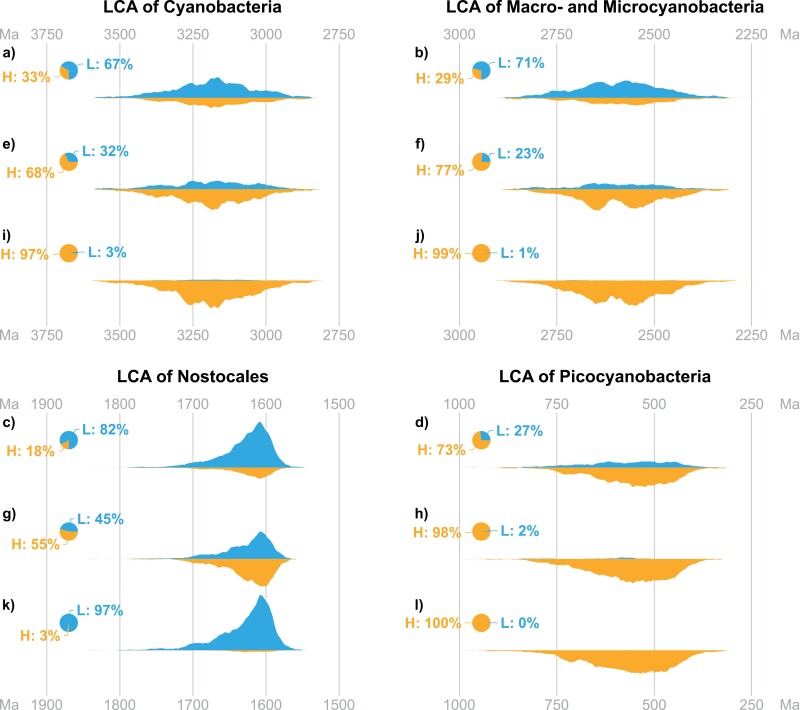
Results of the stochastic mapping analysis for selected nodes. The figure shows the posterior distribution for the age estimate of 4 relevant nodes in our phylogenetic tree of Cyanobacteria (represented by the violin plots as a whole), together with the posterior distribution for the ancestral state of those nodes (represented by the proportion of each violin plot above and below the midline). Plots a, b, c, and d show results of the stochastic mapping analysis with habitat preference as an independent character; e, f, g, and h refer to the analysis with habitat preference conditioned on glucosylglycerol and glucosylglycerate; i, j, k, and l are the results of the analysis with habitat preference conditioned on sucrose, glucosylglycerol, glucosylglycerate, and glycine betaine. For each node, the 3 violin plots (e.g., a, e, and i) show the same distribution for the posterior age estimate, with different ancestral state estimates.

The results of our stochastic mapping analysis, when treating habitat preference as an independent character (Sánchez-Baracaldo 2015; Nakov et al. 2017; Sánchez-Baracaldo et al. 2017b, 2017a) are consistent with previous studies. When considering the ER model, the LCA of Cyanobacteria has a 77% posterior probability of preferring low-salinity habitats and a 23% probability of a preference for high-salinity habitats. Using the ARD model, instead, results in a 55% probability for low-salinity habitat preference and 45% probability of high-salinity habitat preference. The posterior probabilities for the 2 models are, respectively, 55% and 45% (Supplementary Table S8); thus, Bayesian model averaging results in a 67% probability of low-salinity habitat preference for the LCA of Cyanobacteria, and 33% probability of high-salinity habitat preference (Fig. 4a). Results are similarly uncertain for the LCA of Micro- and Macrocyanobacteria, which has a 71% probability of preferring low salinity habitats (Fig. 4b), and the LCA of Picocyanobacteria, which has a 73% probability of a high-salinity habitat preference (Fig. 4c). The LCA of the Nostocales has an 82% probability of a low-salinity habitat preference (Fig. 4d). This is intriguing considering that the vast majority of known Nostocales live in freshwater environments (Sukenik et al. 2009) and a low salinity habitat for this ancestor would be expected.

After identifying GG and GGA as the 2 compatible solutes most significantly correlated with habitat preference, we performed a stochastic mapping analysis where the habitat preference character was conditioned on these 2 compatible solutes. For the LCA of crown group Cyanobacteria, adding the information about GG and GGA has reversed the results, producing a 68% probability that this ancestor had a high salinity habitat preference (Fig. 4e). Similarly, the LCA of Macro- and Microcyanobacteria is now predicted to have a high salinity habitat preference (77%, Fig. 4f); however, in both cases, these probabilities are still not high enough to provide a conclusive answer. The estimate for the LCA of the Nostocales has also become more uncertain in this analysis, with a 45% probability of a low salinity habitat preference (Fig. 4g, down from 82% in the previous analysis). The LCA of Picocyanobacteria, instead, is now predicted to have had a high salinity habitat preference with a high degree of confidence (98%, Fig. 4h).

By including Suc and GB in addition to GG, GGA and habitat preference, our analyses predict that the LCA of crown group Cyanobacteria had a high salinity habitat preference (97%, Fig. 4i). Similarly, the LCA of Macro- and Microcyanobacteria also had a high salinity habitat preference (99%, Fig. 4j), while, as predicted by the initial analysis, the LCA of the Nostocales had a low salinity habitat preference (97%, Fig. 4k). The evidence in favor of the LCA of picocyanobacteria having a high salinity habitat preference has also increased, reaching virtual certainty (100%, Fig. 4l).

### Timeline of Habitat Preference Evolution in Cyanobacteria

**Figure 5.**
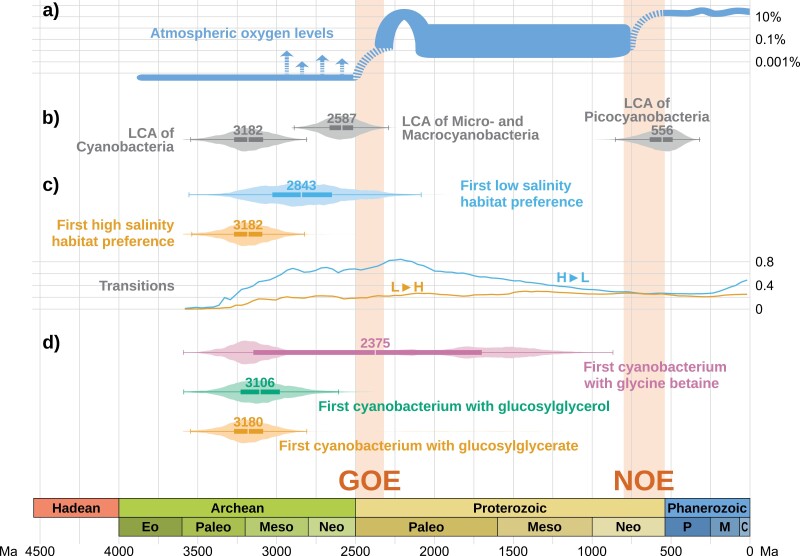
Timeline of major events in the evolution of habitat preference in Cyanobacteria. Age distributions are shown as violin plots with overlaid box plots. The whiskers of the box plot represent 1.5 times the interquartile range (IQR) below the first quartile and 1.5 times the IQR above the third quartile. The value of the median is shown above each box plot. a) Concentration of oxygen expressed as a percentage of the total amount of gas in the atmosphere (adapted from Lyons et al. 2014). b) Posterior age estimates for the LCA of crown-group Cyanobacteria, of Micro- and Macrocyanobacteria, and Picocyanobacteria (respectively, nodes 1, 2, and 4 in Fig. 3). c) Age estimates for the first cyanobacterium with high salinity habitat preference and for the first cyanobacterium with low salinity habitat preference, and transition rates from high to low salinity habitat preference (H►L) and vice versa (L►H), expressed as the average number of transitions per crown group lineage per billion years. d) Age estimates for the first cyanobacterium with glycine betaine, glucosylglycerol, and glucosylglycerate. Note the bimodal distribution for glycine betaine.

In addition to considering specific nodes on the tree, a stochastic mapping analysis makes it possible to analyze when each trait first appeared, regardless of where on the tree this happened. We plotted the posterior distribution for the age estimate of the oldest cyanobacterium with low and high salinity habitat preference (Fig. 5c), together with the age estimates for the LCA of crown-group Cyanobacteria, the LCA of Micro- and Macrocyanobacteria, and the LCA of Picocyanbacteria (Fig. 5b) and a summary of the evolution of atmospheric oxygen levels (Fig. 5a, Lyons et al. 2014). Our analyses suggest that the LCA of crown-group Cyanobacteria had a high salinity habitat preference, therefore the distribution for the oldest cyanobacterium with high salinity habitat preference is almost identical to that of the LCA of Cyanobacteria. Instead, the age distribution for the first cyanobacterium with low salinity habitat preference is much flatter, and this ancestor appears to have evolved later in the history of the phylum, but still during the Archean and close to the GOE. Figure 5c also shows the average number of character state transitions (from low to high salinity habitat preference and vice versa) per lineage per billion years. Throughout the whole history of the phylum, transitions from high to low salinity environments have been more frequent than the opposite. The highest rates are found between approximately 3000 Ma and 1750 Ma, while the transition rate from low salinity to high salinity environments appears to have been lower and consistent throughout time.

As our analyses also suggest the presence of GGA in the cyanobacterial LCA, the age distribution for the first cyanobacterium with GGA (Fig. 5d) is very similar to the age distribution for the LCA of crown-group Cyanobacteria. It is likely that GG was also present in the first cyanobacterium, or that it appeared shortly after it. The pattern for GB presents a bimodal distribution, with a first peak around the origin of LCA of Cyanobacteria, and a second peak much later, between the Paleoproterozoic (2500–1600 Ma) and the Mesoproterozoic (1600–1000 Ma).

## Discussion

### Stochastic Mapping

Our stochastic mapping analyses predict that the LCA of Cyanobacteria had a high-salinity habitat preference, in contrast with previous studies (Sánchez-Baracaldo et al. 2005, 2017b, 2017a; Blank and Sánchez-Baracaldo 2010; Larsson et al. 2011; Latysheva et al. 2012; Dagan et al. 2013; Sánchez-Baracaldo 2015; Uyeda et al. 2016; Hammerschmidt et al. 2021) that estimated a low-salinity ancestral environment for Cyanobacteria. Using different models (e.g., equal-rates or all-rates-different) and priors can have a large effect on the analysis (Nakov et al. 2017; Sánchez-Baracaldo et al. 2017a). The inclusion of models (e.g., the conditioned model, Bianchini and Sánchez‐Baracaldo 2020) that were not available when the previous analyses were conducted is likely one of several factors explaining the disagreement with previous research.

Habitat preference is a complex trait influenced by various environmental and genetic factors. Our initial model testing analyses showed that treating habitat preference as a single morphological character (as done in previous analyses) does not provide enough evidence to address the question of the ancestral state for the LCA of Cyanobacteria. By leveraging the correlation test (*D*-test, Huelsenbeck et al. 2003), the conditioned model, and the dependent model implemented in sMap (Bianchini and Sánchez‐Baracaldo 2020), we are able to consider the genetic information underpinning the biosynthetic pathways of compatible solutes that confer salt tolerance (Mackay et al. 1984; Reed et al. 1986; Scanlan et al. 2009; Hagemann 2011, 2013; Shih et al. 2013), and therefore habitat preference in cyanobacteria. By integrating multiple characters, even where individual characters may not provide enough information in isolation, this new methodology yields results that are highly supported (Fig. 4i–l).

Stochastic mapping uses the specified evolutionary model to generate a sample of possible character histories, constrained to be compatible with the observed data. As is often the case with stochastic mapping analyses (see, e.g., Lemopoulos and Montoya-Burgos 2021; Mehltreter et al. 2022; Corpuz et al. 2023; Larson et al. 2023; Zhang et al. 2023 for some recent examples), previous studies of habitat preference in Cyanobacteria (Sánchez-Baracaldo 2015; Sánchez-Baracaldo et al. 2017b, 2017a) have used these histories to infer ancestral states at the nodes of a phylogenetic tree. In this approach, each ancestral state is assigned a posterior probability corresponding to the proportion of histories in which it is observed, thus producing results that are superficially similar to those obtained through other ancestral state reconstruction methods (Royer-Carenzi et al. 2013). However, the simulated histories also contain much more information, which was used in this study to estimate the timing of events during the evolution of cyanobacteria (Fig. 5c, d), irrespective of the node in the tree at which they occurred. By integrating over all the sampled histories, each of which was simulated on a different tree, we were able to treat the phylogenetic tree as a nuisance parameter, effectively incorporating the uncertainty surrounding the diversification times of the various cyanobacterial clades. This approach can also be applied when the stochastic mapping analysis is conducted using trees with varying topologies (Bianchini and Sánchez‐Baracaldo 2020; Reeves et al. 2023), even though this could not be done in our case because of limitations in the molecular clock model.

A case where information contained in the character histories has relevant implications for the analysis is the timing of the first occurrence of GB in cyanobacteria. Examining the posterior distribution of the event’s age reveals a bimodal distribution that is indicative of 2 contrasting hypotheses. The first hypothesis proposes the presence of GB in the LCA of Cyanobacteria during the Archean, while the second hypothesis posits a significantly later emergence of GB during the Proterozoic. Analyzing a bimodal distribution is challenging, though assessing the 2 alternative hypotheses can be accomplished by comparing how much of the PP density falls within the Archean (>2.5 Ga, 47%) versus the Proterozoic (<2.5 Ga, 53%). The comparison thus indicates that the 2 hypotheses have similar PP. Nevertheless, the scarcity of GB occurrence across cyanobacterial groups (Fig. 3, Hagemann 2011) suggests a relatively delayed acquisition of GB by Cyanobacteria, after much of their diversification had already taken place. In this case, the compatible solute would have originated within a particular group before being transferred to others (e.g., via horizontal gene transfer). The PP for presence of GB in the LCA of Cyanobacteria is 39% (using the conditioned model, or 23% when GB is analyzed independently). These results further highlight the uncertainty surrounding the evolutionary history of this compatible solute in Cyanobacteria, though it is more likely that GB was absent in the LCA of Cyanobacteria.

By analyzing the posterior distribution of the age of the first cyanobacterium with low salinity habitat preference, it is possible to get an estimate that is not constrained to any specific node on the phylogenetic tree. Our results show that there is an 89% probability of this occurring before the GOE at ~2460 Ma (Bekker et al. 2004); these findings imply that although the LCA of Cyanobacteria lived in a high-salinity habitat, low-salinity environments were likely colonized early during the lead-up to the GOE.

Our new findings can also be attributed to the significant influence exerted by GGA and certain GG biosynthesis genes on the analysis; these are predicted to be present in the LCA of cyanobacteria, thereby implying a high salinity ancestral environment. However, it should be noted that GGA itself contributes only slightly to the overall pool of compatible solutes in marine cyanobacteria, which is clearly dominated by GG (Klähn et al. 2010). The negatively charged GGA specifically serves as counterion for K+ in the salt acclimation in habitats with low nitrogen availability, whereas in N-rich environments GGA is usually replaced by glutamate (Klähn et al. 2010 and discussion therein). The pronounced enrichment of GGA in genomes of ancient cyanobacteria may also reflect N-poor conditions. This aligns with other studies that have emphasized the likelihood of nitrogen being a limiting nutrient in the early oceans (Kasting and Siefert 2001; Navarro-González et al. 2001; Stüeken et al. 2016), thus creating a niche that early nitrogen-fixing cyanobacteria could occupy (Latysheva et al. 2012).

It is worth noting that in our analyses we sample 189 taxa to perform inferences about events that happened billions of years ago. While we carefully selected taxa to represent the diversity of modern cyanobacterial habitat preferences, it is important to note that the number of lineages represented in the phylogenetic tree necessarily decreases as we approach the root. This reduction in lineage representation near the root may introduce uncertainty that is not fully accounted by the models (O’meara and Beaulieu 2021). While further sampling and genome sequencing efforts may be able to provide a broader understanding of the analyzed characters, the lack of resolution in older nodes is an inherent characteristic of any phylogenetic method reconstructing past diversity based on present data (O’meara and Beaulieu 2021). This may contribute to the differences between our results and previous analyses. Furthermore, like in most ancestral state reconstruction analyses, model parameters (e.g., transition rates and conditioned probabilities) are assumed to be constant through time along all lineages, introducing further uncertainty (Cunningham et al. 1998). The assumption of constant model parameters is necessary due to the large number of parameters that would need to be estimated if lineage-specific rates were allowed. A rooted binary phylogenetic tree with *n* taxa contains a total of 2*n*−2 branches, and allowing lineage-specific rates would thus require estimating a large number of parameters from a single observation for each taxon. This issue does not arise for DNA or amino acid sequences, as many sequence sites are assumed to evolve under the same model. In summary, though our analyses predict a high-salinity ancestral environment for Cyanobacteria with high posterior probability, it is important to consider that our results are conditioned on the observed data, the models we considered, and the software we employed, and thus subject to all their caveats.

### Model Analysis

We employed marginal likelihoods whenever possible to select the optimal model and to compute mPPs in order to perform Bayesian model averaging. For the models used in our final analysis, computing exact marginal likelihoods was not feasible, as it would have required an excessive amount of computation time. Therefore, we used approximate marginal likelihoods based on the results obtained for individual characters. An alternative to this approach would have involved using a maximum-likelihood information criterion such as AIC or BIC (Akaike 1974; Schwarz 1978), which could be then transformed into the corresponding model weights (Wagenmakers and Farrell 2004) for the purpose of model averaging.

The AIC, BIC, and marginal likelihood/mPP approaches have different assumptions and objectives. The AIC aims to minimize information loss when approximating reality with a simplified model (Akaike 1974). On the other hand, the BIC aims to select the “true model” that generated the data, which is assumed to be between the ones being analyzed (Schwarz 1978). Conversely, mPPs estimate the support in favor of each model, given the data and a prior distribution over the models and model parameters (Xie et al. 2011). Furthermore, the BIC and AIC use a point estimate, i.e., the maximum-likelihood estimate, to compare the models, while marginal likelihoods and mPPs measure an average over the whole parameter space, weighted by the priors on parameter values (Xie et al. 2011). In our specific case, despite these differences, the approximate mPPs that we used for the final analysis were very similar to the AIC weights (Supplementary Table S10). If we were to instead use the BIC weights, some results would become slightly more uncertain (e.g., the posterior probability for high salinity preference in the LCA of Cyanobacteria would become 79% rather than 97%), but the same conclusions would be supported.

### Transition Rates

The transition rate parameters within a character model provide information about evolutionary patterns. For instance, the loss/gain (*L*/*G*) rate ratio in our model highlights the overall trend to acquire or lose compatible solutes across the entire phylogenetic tree. Among the 5 compatible solutes analyzed, *L*/*G* ratios are higher than 1 for GG, GGA, and GB, and much smaller for Suc and Tre. This discrepancy likely arises from the fact that while cyanobacteria predominantly employ GG, GGA, and GB as compatible solutes, Suc and Tre fulfill additional functions within cells. For example, sucrose often serves as carbon skeletons transported from vegetative cells into N_2_-fixing heterocysts (Kolman et al. 2015), while trehalose is particularly important for desiccation tolerance (Klähn and Hagemann 2011). Notably, in this analysis, we did not differentiate between terrestrial and freshwater environments. Suc and Tre can be thus regarded as “last resort” compatible solutes (Hagemann 2013). Therefore, it is likely that different compatible solutes are subject to distinct selective pressures resulting in different patterns of evolution (i.e., differences in the balance between gains and losses between compatible solutes).

In neutral conditions, gene loss rates are generally greater than gain rates (Mira et al. 2001; Kuo and Ochman 2009). For instance, losing an enzyme-coding gene is a relatively easy process, which can be kick-started by a single nucleotide substitution affecting the active site (Mira et al. 2001; Kuo and Ochman 2009). In contrast, the acquisition of a new gene involves a number of concomitant conditions. A lateral gene transfer event requires, for example, the donor strain and receiving strain to be present and close at the same time, the receiving strain to be competent, the nucleotide sequence to integrate into the genome, the gene to be expressed in the right conditions, and so on (Ochman et al. 2000). This effect can however be balanced or outweighed if the gene provides a significant selective advantage once it has been gained (Mira et al. 2001), which appears to be the case for Tre and Suc.

Some metabolisms have intricated evolutionary histories. Suc is particularly complicated to analyze, because the C terminus of *spsA* is a homolog of *spp* (Martínez-Noël et al. 2013), and it is possible that Suc is produced using a single gene for both steps of the pathway (Hagemann 2013); thus, in principle, both *spsA* on its own and spsA* with *spp* can also correspond to a complete pathway. This situation is particularly difficult among *Prochlorococcus* spp., in which only some strains harbor a complete *spsA* gene, while the majority has truncated *spsA* genes without the 3ʹ end encoding the *spp* part. At the same time, no separate *spp* genes are present in *Prochlorococcus* genomes. It is tempting to speculate that the frequently occurring *ggpP* genes might have acquired sucrose-phosphate phosphatase activity in addition to their ancestral GG-phosphate phosphatase activity, thus supporting sucrose accumulation. Assuming that a complete Suc pathway may be inferred by the presence of *spsA* (with or without *spp*) or of both spsA* and *spp*, losses of Suc are (slightly) less likely than gains (Supplementary Fig. S14), with Suc being the most widespread compatible solute across cyanobacteria (Fig. 3, Hagemann 2011). This is consistent with Suc having other fundamental roles within cyanobacteria, such as being involved in nitrogen fixation and carbon metabolism (Lunn 2002; Cumino et al. 2007; Kolman et al. 2012).

In the final analysis, the habitat preference character is not modeled using a Markov model; as a result, the model does not include rate parameters for this character. Stochastic mapping simulations can be used to estimate the relative transition rates between habitats by plotting the (normalized) number of transitions between high salinity and low salinity habitat preference over time (Fig. 5c). Our analyses reveal that the average transition rate from L→H (i.e., $r_{L\to H}$) has remained relatively stable over the past 3 billion years. In contrast, $r_{H\to L}$ has been more variable. In particular, $r_{H\to L}$ has always been higher than $r_{L\to H}$, though the difference between the 2 is greatest between the end of the Archean and the Paleoproterozoic. During this period, there were up to 4 times as many transitions from high salinity to low salinity habitat preference compared to the reverse direction. The intensity and mechanics of plate tectonics in the early Earth are still the subject of debate (Brown et al. 2020), but these results may be consistent with the appearance of new low salinity environments, during a time of geological upheaval resulting from the establishment of modern plate tectonics.

Comparing the transition rates suggests that it is easier for a strain living in a high-salinity environment to adapt to a low-salinity environment, than vice versa. From a biological point of view, this is consistent with previous observations in which many salt-tolerant cyanobacteria (i.e., strains that are able to tolerate salt concentrations higher than or equal to seawater) are capable of growing in low-salinity media. In contrast, freshwater strains are not able to grow in high-salinity media (Mackay et al. 1984; Reed et al. 1986). This highlights that cells containing the genetic machinery needed to deal with the osmotic pressures in a high-salinity environment can stop expressing compatible solutes (e.g., GG, GGA) when moved into a low-salinity environment (Scanlan et al. 2009; Hagemann 2013; Shih et al. 2013). Moving in the opposite direction, freshwater to marine, would however require the bacterium to develop a new biochemical pathway. An example of this is the strain *Nostoc ellipsosporum* NOK that has recently acquired a *ggpPS* gene homolog (MDF2386645.1) that encodes for a fusion protein capable of GG-phosphate synthase and phosphatase reactions (Hagemann et al. 2008), whereas in all other *Nostoc spp.* no GG synthesis genes are present. Many cyanobacterial strains that produce compatible solutes, in fact, are able to regulate their biosynthesis, so that they can survive in both high- and low-salinity conditions (Scanlan et al. 2009; Hagemann 2013; Shih et al. 2013).

Finally, $r_{H\to L}$ is highest during the Paleoproterozoic, shortly after the GOE. This reflects a period of high diversification for cyanobacteria (Schirrmeister et al. 2013), as shown also by the many groups that originate around this time (Boden et al. 2021; Fournier et al. 2021). This is consistent with the colonization of new habitats (Blank and Sánchez-Baracaldo 2010) and the ecological success of cyanobacteria during the rest of the Precambrian, as reflected by the fossil record (Schirrmeister et al. 2016).

## Conclusions

The origins of oxygenic photosynthesis, the LCA of Cyanobacteria, and the ecological habitats in which these organisms evolved are among the most fundamental questions regarding the emergence of the Earth’s biosphere. Cyanobacteria were the only prokaryotes to evolve oxygenic photosynthesis and thus played a key role by changing the redox surface of the planet and fueling aerobic metabolisms (Lyons et al. 2014; Sánchez-Baracaldo et al. 2022). Age estimates for the LCA of Cyanobacteria continue to be debated, partly due to substantial uncertainty in the geological record (e.g., geochemistry and microfossils) of the Archean (Farquhar et al. 2011; Pufahl and Hiatt 2012; Schirrmeister et al. 2016). Phylogenomic and relaxed molecular clock analyses have shed light into the timing of the origin of the photosynthetic reaction center core proteins possessed by oxygenic phototrophs (Cardona et al. 2019; Oliver et al. 2021), their divergence from their closest non-photosynthetic relatives (Soo et al. 2014; Boden et al. 2021) and their LCA (Fournier et al. 2021). In the last decade, the increased availability of sequenced bacterial genomes has improved the resolution of phylogenetic relationships both within Cyanobacteria, as well as between them and their closest Terrabacteria relatives (Coleman et al. 2021). In this study, we demonstrate how information from cyanobacterial genomes is a valuable resource for investigating the evolution of mechanisms underlying their habitat preferences.

Our stochastic mapping analyses suggest that the LCA of Cyanobacteria likely originated in marine environments around 3180 Ma, which aligns with geochemical evidence indicating the presence of oxygen-mobilized trace elements (e.g., Mo, Cr, Re, U) in some Archean deposits as early as 3000 Ma (Lyons et al. 2014). It is important to note that while uncertainties exist regarding the specific conditions of the Archean environment (e.g., temperature, pH, pCO_2_), the existence of liquid water and carbonate precipitation in the early rock record indicates that ocean temperatures and pH were not significantly different from today (Grotzinger and Kasting 1993; Arndt and Nisbet 2012). Salt tolerance in cyanobacterial stem groups is consistent with the presence of organic-rich sedimentary rocks, such as black shales, which indicate the accumulation of organic carbon in ancient oceans prior to the GOE (Lyons et al. 2014; Knoll et al. 2016). These shales often contain high concentrations of organic matter, suggesting the existence of productive ecosystems and the burial of organic carbon presumably by oxygenic phototrophs (Lyons et al. 2014). While the abiotic conditions of the Proterozoic ocean in which cyanobacteria emerged remain uncertain, there is evidence to suggest that seawater chemistry and composition have changed over time with an increase in abundance of ionic solutes (e.g., magnesium, calcium, and sulfate ions) (Habicht et al. 2002; Crowe et al. 2014; Turchyn and Depaolo 2019). Despite an age estimate of ~3180 Ma for the LCA of cyanobacteria, it still took more than 700 Myr for the GOE to take place. Late Archean marine carbonate sediments show similar carbon isotope compositions to most of the Proterozoic and Phanerozoic ones suggesting that the carbon cycle was burying similar proportions of organic carbon, presumably as a result mostly of oxygenic phototrophs, before the rise of atmospheric oxygen (Farquhar et al. 2011; Knoll et al. 2016). Although several biogeochemical hypotheses have explained controls on primary productivity (e.g., toxic effects of ferrous iron in solution on rates of oxygenic photosynthesis, Swanner et al. 2015, lack of phosphorus availability, Alcott et al. 2022; Jones et al. 2015) few studies have looked at specific cyanobacterial groups and environments that may have contributed to marine primary productivity (Sánchez-Baracaldo et al. 2014). Moreover, little attention has been given to the evolutionary processes resulting in increased cyanobacteria diversification over time and their consequences for biogeochemical cycles (e.g., C, N, O) (Sánchez-Baracaldo et al. 2022).

Trait evolution analyses indicate that the earliest branching cyanobacteria were likely unicellular organisms (Blank and Sánchez-Baracaldo 2010; Larsson et al. 2011; Sánchez-Baracaldo 2015). During the late Archean period, there was an evolutionary transition toward filamentous forms with stem *Pseudanabaena* lineages diverging near the root of the cyanobacterial tree (Sánchez-Baracaldo 2015). This transition to filamentous growth was a significant biological innovation that facilitated the formation of microbial mats and biofilms, contributing to the ecological success of cyanobacteria (Blank and Sánchez-Baracaldo 2010). Our analyses suggest that freshwater cyanobacteria ancestors emerged around 2800 Ma, indicating that by this time, cyanobacteria had already colonized a wide range of habitats, including terrestrial both low- and high-salinity aquatic environments (Lalonde and Konhauser 2015; Sánchez-Baracaldo et al. 2022). Previous studies have proposed that the evolution of multicellularity in cyanobacteria played a role in increased diversification rates around the GOE (Schirrmeister et al. 2013, 2016), potentially facilitating the ecological expansion of cyanobacteria during this period.

While the geological record provides evidence of widespread cyanobacterial mats throughout the Proterozoic, reflecting the early diversification of filamentous lineages (Bosak et al. 2013), it has been less clear which cyanobacterial groups appeared around the time of the GOE. Our age estimate for the last common ancestor of the Macro- and Microcyanobacteria clades (~2590 Ma) suggests that these 2 clades were likely major contributors to primary productivity in marine habitats leading up to the GOE. Previous works have shown that these clades also present biological innovations associated with the formation of thick, laminated microbial mats (Blank and Sánchez-Baracaldo 2010) such as an increase in cell diameter in the ancestors of Macrocyanobacteria (Sánchez-Baracaldo 2015), sheaths, and motility amongst others (Sánchez-Baracaldo et al. 2005; Blank and Sánchez-Baracaldo 2010; Sánchez-Baracaldo 2015). The ecological expansion of marine cyanobacterial ancestors in shallow marine environments would have allowed for significant burial of organic carbon in anoxic marine waters, leading to the gradual accumulation of oxygen over thousands of years (Hayes and Waldbauer 2006; Blank and Sánchez-Baracaldo 2010). We thus propose that the divergence of Macro- and Microcyanobacteria played a pivotal role in triggering the sudden rise in atmospheric oxygen.

## Supplementary Material

Data available from the Dryad Digital Repository: https://dx.doi.org/10.5061/dryad.bnzs7h4hq

## Data Availability

Supplementary materials, including the data underlying this article, can be found in the Dryad data repository at https://dx.doi.org/10.5061/dryad.bnzs7h4hq. These include sequence alignments, phylogenetic tree files, and source code for the scripts used in the article.

## References

[CIT0001] Akaike H. 1974. A new look at the statistical model identification. IEEE Trans. Automat. Contr. 19:716–723. doi: 10.1109/TAC.1974.1100705

[CIT0002] Alcott L.J. , Mills B.J.W., Bekker A., Poulton S.W. 2022. Earth’s great oxidation event facilitated by the rise of sedimentary phosphorus recycling. Nat. Geosci. 15:210–215. doi: 10.1038/s41561-022-00906-5

[CIT0003] Arndt N.T. , Nisbet E.G. 2012. Processes on the young earth and the habitats of early life. Annu. Rev. Earth Planet. Sci. 40:521–549. doi: 10.1146/ANNUREV-EARTH-042711-105316

[CIT0139] Badger M.R , Hanson D, Price G.D. 2002. Evolution and diversity of CO2 concentrating mechanisms in cyanobacteria. Functional Plant Biology 29(3):161–161. doi:10.1071/PP01213 32689463

[CIT0004] Battistuzzi F.U. , Feijao A., Hedges S.B. 2004. A genomic timescale of prokaryote evolution: insights into the origin of methanogenesis, phototrophy, and the colonization of land. BMC Evol. Biol. 4:44. doi: 10.1186/1471-2148-4-44 15535883 PMC533871

[CIT0005] Battistuzzi F.U. , Hedges S.B. 2009. A major clade of prokaryotes with ancient adaptations. Mol. Biol. Evol. 26:335–343. doi: 10.1093/molbev/msn247 18988685

[CIT0006] Bekker A. , Holland H.D., Wang P.-L., Rumble D., Stein H.J., Hannah J.L., Coetzee L.L., Beukes N.J. 2004. Dating the rise of atmospheric oxygen. Nature 427:117–120. doi: 10.1038/nature02260 14712267

[CIT0007] Bianchini G. 2017. AlignmentViewer. Available from: URL https://giorgiobianchini.com/AlignmentViewer/AlignmentViewer.html

[CIT0008] Bianchini G. 2019. arklumpus/VectSharp: A light library for C# vector graphics. Available from: URL https://github.com/arklumpus/VectSharp

[CIT0009] Bianchini G. 2023. TreeNode: Version 1.5.3. Available from: URL 10.5281/ZENODO.8387417

[CIT0010] Bianchini G. , Sánchez-Baracaldo P. 2024. TreeViewer: flexible, modular software to visualise and manipulate phylogenetic trees. Ecol. Evol. 14:e10873. doi: 10.1002/ECE3.10873 38314311 PMC10834882

[CIT0011] Bianchini G. , Sánchez‐Baracaldo P. 2020. sMap: Evolution of independent, dependent and conditioned discrete characters in a Bayesian framework. Methods Ecol. Evol. 12:479–486. doi: 10.1111/2041-210X.13540

[CIT0012] Blank C.E. , Sánchez-Baracaldo P. 2010. Timing of morphological and ecological innovations in the cyanobacteria—a key to understanding the rise in atmospheric oxygen. Geobiology 8:1–23. doi: 10.1111/j.1472-4669.2009.00220.x 19863595

[CIT0013] Boden J.S. , Konhauser K.O., Robbins L.J., Sánchez-Baracaldo P. 2021. 2021. Timing the evolution of antioxidant enzymes in cyanobacteria. Nat. Commun. 12:1. 12:1–12. doi: 10.1038/s41467-021-24396-y 34362891 PMC8346466

[CIT0014] Bosak T. , Knoll A.H., Petroff A.P. 2013. The meaning of stromatolites. Annu. Rev. Earth Planet. Sci. 41:21–44. doi: 10.1146/ANNUREV-EARTH-042711-105327

[CIT0015] Brown A.D. 1976. Microbial water stress. Bacteriol Rev 40:803–846. 1008746 10.1128/br.40.4.803-846.1976 PMC413986

[CIT0016] Brown M. , Johnson T., Gardiner N.J. 2020. Plate tectonics and the Archean Earth. Annu. Rev. Earth Planet. Sci. 48:291–320. doi: 10.1146/ANNUREV-EARTH-081619-052705

[CIT0017] Camacho C. , Coulouris G., Avagyan V., Ma N., Papadopoulos J., Bealer K., Madden T.L. 2009. BLAST+: architecture and applications. BMC Bioinf. 10:421. doi: 10.1186/1471-2105-10-421 PMC2803857 20003500

[CIT0018] Cardona T. , Sánchez-Baracaldo P., Rutherford A.W., Larkum A.W. 2019. Early Archean origin of photosystem II. Geobiology 17:127–150. doi: 10.1111/gbi.12322 30411862 PMC6492235

[CIT0019] Castenholz R.W. , Wilmotte A., Herdman M., Rippka R., Waterbury J.B., Iteman I., Hoffmann L. 2001. Phylum BX. Cyanobacteria. In Boone, D.R., Castenholz, R.W., Garrity, G.M., editors. Bergey’s manual® of systematic bacteriology. New York, NY: Springer. p. 473–599 doi: 10.1007/978-0-387-21609-6_27

[CIT0020] Chen M.Y. , Teng W.K., Zhao L., Hu C.X., Zhou Y.K., Han B.P., Song L.R., Shu W.S. 2021. Comparative genomics reveals insights into cyanobacterial evolution and habitat adaptation. ISME J. 15:211–227. doi: 10.1038/S41396-020-00775-Z 32943748 PMC7852516

[CIT0021] Chen T.H.H. , Murata N. 2011. Glycinebetaine protects plants against abiotic stress: mechanisms and biotechnological applications. Plant Cell Environ. 34:1–20. doi: 10.1111/j.1365-3040.2010.02232.x 20946588

[CIT0022] Clark K. , Karsch-Mizrachi I., Lipman D.J., Ostell J., Sayers E.W. 2016. GenBank. Nucleic Acids Res. 44:D67–D72. doi: 10.1093/NAR/GKV1276 26590407 PMC4702903

[CIT0023] Coleman G.A. , Davín A.A., Mahendrarajah T.A., Szánthó L.L., Spang A., Hugenholtz P., Szöllsi G.J., Williams T.A. 2021. A rooted phylogeny resolves early bacterial evolution. Science (1979) 372:eabe0511. doi:10.1126/SCIENCE.ABE0511 33958449

[CIT0024] Corpuz R.L. , Bellinger M.R., Veillet A., Magnacca K.N., Price D.K. 2023. The Transmission patterns of the endosymbiont *Wolbachia* within the Hawaiian Drosophilidae adaptive radiation. Genes (Basel) 14:1545. doi: 10.3390/GENES14081545 37628597 PMC10454618

[CIT0025] Costa J. , Empadinhas N., Gonçalves L., Lamosa P., Santos H., da Costa M.S. 2006. Characterization of the biosynthetic pathway of glucosylglycerate in the archaeon *Methanococcoides burtonii*. J. Bacteriol. 188:1022–1030. doi: 10.1128/JB.188.3.1022-1030.2006 16428406 PMC1347341

[CIT0026] Crowe S.A. , Paris G., Katsev S., Jones C.A., Kim S.T., Zerkle A.L., Nomosatryo S., Fowle D.A., Adkins J.F., Sessions A.L., Farquhar J., Canfield D.E. 2014. Sulfate was a trace constituent of Archean seawater. Science (New York, N.Y.) 346:735–739. doi: 10.1126/SCIENCE.1258966 25378621

[CIT0027] Cumino A.C. , Marcozzi C., Barreiro R., Salerno G.L. 2007. Carbon cycling in Anabaena sp. PCC 7120. Sucrose synthesis in the heterocysts and possible role in nitrogen fixation. Plant Physiol. 143:1385–1397. doi: 10.1104/PP.106.091736 17237189 PMC1820908

[CIT0028] Cunningham C.W. , Omland K.E., Oakley T.H. 1998. Reconstructing ancestral character states: a critical reappraisal. Trends Ecol. Evol. 13:361–366. doi: 10.1016/S0169-5347(98)01382-2 21238344

[CIT0029] Dagan T. , Roettger M., Stucken K., Landan G., Koch R., Major P., Gould S.B., Goremykin V.V., Rippka R., De Marsac N.T., Gugger M., Lockhart P.J., Allen J.F., Brune I., Maus I., Pühler A., Martin W.F. 2013. Genomes of stigonematalean cyanobacteria (Subsection V) and the evolution of oxygenic photosynthesis from prokaryotes to plastids. Genome Biol. Evol. 5:31–44. doi: 10.1093/GBE/EVS117 23221676 PMC3595030

[CIT0030] Demoulin C.F. , Lara Y.J., Cornet L., François C., Baurain D., Wilmotte A., Javaux E.J. 2019. Cyanobacteria evolution: Insight from the fossil record. Free Radic. Biol. Med. 140:206–223. doi: 10.1016/j.freeradbiomed.2019.05.007 31078731 PMC6880289

[CIT0031] di Rienzi S.C. , Sharon I., Wrighton K.C., Koren O., Hug L.A., Thomas B.C., Goodrich J.K., Bell J.T., Spector T.D., Banfield J.F., Ley R.E. 2013. The human gut and groundwater harbor non-photosynthetic bacteria belonging to a new candidate phylum sibling to cyanobacteria. Elife 2:e01102. doi:10.7554/eLife.01102.001 24137540 PMC3787301

[CIT0032] Díez B. , Ininbergs K. 2013. Ecological importance of cyanobacteria. Cyanobacteria. Chichester, UK: John Wiley & Sons, Ltd. p. 41–63 doi: 10.1002/9781118402238.ch3

[CIT0033] Farquhar J. , Zerkle A.L., Bekker A. 2011. Geological constraints on the origin of oxygenic photosynthesis. Photosynth. Res. 107:11–36. doi: 10.1007/S11120-010-9594-0 20882345

[CIT0034] Felsenstein J. 1985. Phylogenies and the comparative method. Am. Nat. 125:1–15. doi: 10.1086/284325

[CIT0035] Fourment M. , Magee A.F., Whidden C., Bilge A., Matsen F.A., Minin V.N. 2020. 19 dubious ways to compute the marginal likelihood of a phylogenetic tree topology. Syst. Biol. 69:209–220. doi: 10.1093/SYSBIO/SYZ046 31504998 PMC7571498

[CIT0036] Fournier G.P. , Moore K.R., Rangel L.T., Payette J.G., Momper L., Bosak T. 2021. The Archean origin of oxygenic photosynthesis and extant cyanobacterial lineages. Proc. Royal Soc. B 288:20210675. doi: 10.1098/RSPB.2021.0675 PMC8479356 34583585

[CIT0037] Fragoso T.M. , Bertoli W., Louzada F. 2018. Bayesian model averaging: a systematic review and conceptual classification. Int. Stat. Rev. 86:1–28. doi: 10.1111/INSR.12243

[CIT0038] Garcia-Pichel F. , Zehr J.P., Bhattacharya D., Pakrasi H.B. 2020. What’s in a name? The case of cyanobacteria. J. Phycol. 56:1–5. doi: 10.1111/JPY.12934 31618454 PMC7065140

[CIT0039] Grotzinger J.P. , Kasting J.F. 1993. New constraints on Precambrian ocean composition. J. Geol. 101:235–243. doi: 10.1086/648218 11537740

[CIT0040] Guida B.S. , Garcia-Pichel F. 2016. Draft genome assembly of a filamentous euendolithic (True Boring) cyanobacterium, mastigocoleus testarum strain BC008. Genome Announc. 4:e01574–e01515. doi: 10.1128/GENOMEA.01574-15 26823575 PMC4732328

[CIT0041] Habicht K.S. , Gade M., Thamdrup B., Berg P., Canfield D.E. 2002. Calibration of sulfate levels in the Archean ocean. Science (New York, N.Y.) 298:2372–2374. doi: 10.1126/SCIENCE.1078265 12493910

[CIT0042] Hagemann M. 2011. Molecular biology of cyanobacterial salt acclimation. FEMS Microbiol. Rev. 35:87–123. doi: 10.1111/j.1574-6976.2010.00234.x 20618868

[CIT0043] Hagemann M. 2013. Genomics of salt acclimation: synthesis of compatible solutes among cyanobacteria. Adv. Bot. Res. 65:27–55. doi: 10.1016/B978-0-12-394313-2.00002-0

[CIT0044] Hagemann M. , Ribbeck-Busch K., Klähn S., Hasse D., Steinbruch R., Berg G. 2008. The plant-associated bacterium *Stenotrophomonas rhizophila* expresses a new enzyme for the synthesis of the compatible solute glucosylglycerol. J. Bacteriol. 190:5898–5906. doi: 10.1128/JB.00643-08 18586931 PMC2519522

[CIT0045] Hammerschmidt K. , Landan G., Domingues Kümmel Tria F., Alcorta J., Dagan T. 2021. The order of trait emergence in the evolution of cyanobacterial multicellularity. Genome Biol. Evol. 13:evaa249. doi: 10.1093/GBE/EVAA249 33231627 PMC7937182

[CIT0046] Hayes J.M. , Waldbauer J.R. 2006. The carbon cycle and associated redox processes through time. Philos. Trans. R. Soc. London, Ser. B 361:931–950. doi: 10.1098/RSTB.2006.1840 16754608 PMC1578725

[CIT0138] Hess W.R. , Rocap G., Ting C.S., Larimer F., Stilwagen S., Lamerdin J., Chisholm S.W. 2001. The photosynthetic apparatus of Prochlorococcus: Insights through comparative genomics. Photosynthesis Research, 70(1):53–71. doi: 10.1023/A:1013835924610 16228362

[CIT0047] Hoeting J.A. , Madigan D., Raftery A.E., Volinsky C.T. 1999. Bayesian model averaging: a tutorial. Stat. Sci. 14:382–401. doi: 10.1214/SS/1009212519

[CIT0048] Hoffmann L. , Komárek J., Kaštovský J. 2005. System of cyanoprokaryotes (cyanobacteria) – state in 2004. Arch Hydrobiol Suppl Algol Stud 117:95–115. doi: 10.1127/1864-1318/2005/0117-0095

[CIT0049] Huelsenbeck J.P. , Bollback J.P. 2001. Empirical and hierarchical Bayesian estimation of ancestral states. Syst. Biol. 50:351–366. 12116580

[CIT0050] Huelsenbeck J.P. , Nielsen R., Bollback J.P. 2003. Stochastic mapping of morphological characters. Syst. Biol. 52:131–158. doi: 10.1080/10635150390192780 12746144

[CIT0051] Huey R.B. , Bennett A.F. 1987. Phylogenetic studies of coadaptation: preferred temperatures versus optimal performance temperatures of lizards. Evolution Int. J. Org Evolution 41:1098–1115. doi: 10.1111/j.1558-5646.1987.tb05879.x 28563407

[CIT0052] The Inkscape Project. 2003. Inkscape. Available from: URL https://inkscape.org/

[CIT0053] Jasser I. , Callieri C. 2017. Picocyanobacteria. In Meriluoto, J., Spoof, L., Codd, G.A., editors. Handbook of cyanobacterial monitoring and cyanotoxin analysis. Chichester, West Sussex, United Kingdom: John Wiley & Sons, Ltd. p. 19–27. doi: 10.1002/9781119068761.CH3

[CIT0054] Jones C. , Nomosatryo S., Crowe S.A., Bjerrum C.J., Canfield D.E. 2015. Iron oxides, divalent cations, silica, and the early earth phosphorus crisis. Geology 43:135–138. doi: 10.1130/G36044.1

[CIT0055] Kalyaanamoorthy S. , Minh B.Q., Wong T.K.F., von Haeseler A., Jermiin L.S. 2017. ModelFinder: fast model selection for accurate phylogenetic estimates. Nat. Methods 14:587–589. doi: 10.1038/nmeth.4285 28481363 PMC5453245

[CIT0056] Kasting J.F. , Siefert J.L. 2001. The nitrogen fix. Nature 412:26–27. doi: 10.1038/35083660 11452283

[CIT0057] Katoh K. , Standley D.M. 2013. MAFFT multiple sequence alignment software version 7: improvements in performance and usability. Mol. Biol. Evol. 30:772–780. doi: 10.1093/molbev/mst010 23329690 PMC3603318

[CIT0058] Klähn S. , Hagemann M. 2011. Compatible solute biosynthesis in cyanobacteria. Environ. Microbiol. 13:551–562. doi: 10.1111/J.1462-2920.2010.02366.X 21054739

[CIT0059] Klähn S. , Steglich C., Hess W.R., Hagemann M. 2010. Glucosylglycerate: a secondary compatible solute common to marine cyanobacteria from nitrogen-poor environments. Environ. Microbiol. 12:83–94. doi: 10.1111/J.1462-2920.2009.02045.X 19735283

[CIT0060] Klebanov I. , Sikorski A., Schütte C., Röblitz S. 2021. Objective priors in the empirical Bayes framework. Scand. J. Stat. 48:1212–1233. doi: 10.1111/SJOS.12485

[CIT0061] Knoll A.H. 2012. The fossil record of microbial life. Fundamentals of geobiology. Chichester, UK: John Wiley & Sons, Ltd. p. 297–314 doi: 10.1002/9781118280874.ch16

[CIT0062] Knoll A.H. , Bergmann K.D., Strauss J.V. 2016. Life: the first two billion years. Philos. Trans. Royal Soc. B: Biol. Sci. 371:20150493. doi: 10.1098/RSTB.2015.0493 PMC5052739 27672146

[CIT0063] Kolman M.A. , Nishi C.N., Perez-Cenci M., Salerno G.L. 2015. Sucrose in cyanobacteria: from a salt-response molecule to play a key role in nitrogen fixation. Life (Basel) 5:102–126. doi: 10.3390/LIFE5010102 25569239 PMC4390843

[CIT0064] Kolman M.A. , Torres L.L., Martin M.L., Salerno G.L. 2012. Sucrose synthase in unicellular cyanobacteria and its relationship with salt and hypoxic stress. Planta 235:955–964. doi: 10.1007/s00425-011-1542-5 22113826

[CIT0065] Komárek J. , Kaštovský J., Mareš J., Johansen J.R. 2014. Taxonomic classification of cyanoprokaryotes (cyanobacterial genera) 2014, using a polyphasic approach. Preslia 86:295–335.

[CIT0066] Kuo C.-H. , Ochman H. 2009. Deletional bias across the three domains of life. Genome Biol. Evol. 1:145–152. doi: 10.1093/GBE/EVP016 20333185 PMC2817411

[CIT0067] Lalonde S.V. , Konhauser K.O. 2015. Benthic perspective on Earth’s oldest evidence for oxygenic photosynthesis. Proc. Natl. Acad. Sci. U. S. A. 112:995–1000. doi: 10.1073/PNAS.1415718112 25583484 PMC4313849

[CIT0068] Larson D.A. , Chanderbali A.S., Maurin O., Gonçalves D.J.P., Dick C.W., Soltis D.E., Soltis P.S., Fritsch P.W., Clarkson J.J., Grall A., Davies N.M.J., Larridon I., Kikuchi I.A.B.S., Forest F., Baker W.J., Smith S.A., Utteridge T.M.A. 2023. The phylogeny and global biogeography of Primulaceae based on high-throughput DNA sequence data. Mol. Phylogenet. Evol. 182:107702. doi: 10.1016/J.YMPEV.2023.107702 36781032

[CIT0069] Larsson J. , Nylander J.A.A., Bergman B. 2011. Genome fluctuations in cyanobacteria reflect evolutionary, developmental and adaptive traits. BMC Evol. Biol. 11:1–21. doi: 10.1186/1471-2148-11-187 21718514 PMC3141437

[CIT0070] Lartillot N. , Philippe H. 2004. A Bayesian mixture model for across-site heterogeneities in the amino-acid replacement process. Mol. Biol. Evol. 21:1095–1109. doi: 10.1093/molbev/msh112 15014145

[CIT0071] Lartillot N. , Philippe H. 2006. Computing Bayes factors using thermodynamic integration. Syst. Biol. 55:195–207. doi: 10.1080/10635150500433722 16522570

[CIT0072] Latysheva N. , Junker V.L., Palmer W.J., Codd G.A., Barker D. 2012. The evolution of nitrogen fixation in cyanobacteria. Bioinformatics 28:603–606. doi: 10.1093/BIOINFORMATICS/BTS008 22238262

[CIT0073] Lemopoulos A. , Montoya-Burgos J.I. 2021. From scales to armor: scale losses and trunk bony plate gains in ray-finned fishes. Evol. Lett. 5:240–250. doi: 10.1002/EVL3.219 34136272 PMC8190451

[CIT0074] Logares R. , Bråte J., Bertilsson S., Clasen J.L., Shalchian-Tabrizi K., Rengefors K. 2009. Infrequent marine–freshwater transitions in the microbial world. Trends Microbiol. 17:414–422. doi: 10.1016/J.TIM.2009.05.010 19726194

[CIT0075] Lunn J.E. 2002. Evolution of sucrose synthesis. Plant Physiol. 128:1490–1500. doi: 10.1104/pp.010898 11950997 PMC154276

[CIT0076] Lyons T.W. , Reinhard C.T., Planavsky N.J. 2014. The rise of oxygen in Earth’s early ocean and atmosphere. Nature 506:307–315. doi: 10.1038/nature13068 24553238

[CIT0077] Mackay M.A. , Norton R.S., Borowitzka L.J. 1984. Organic osmoregulatory solutes in cyanobacteria. Microbiology 130:2177–2191. doi: 10.1099/00221287-130-9-2177

[CIT0078] Maddison W.P. 1990. A method for testing the correlated evolution of two binary characters are gains or losses concentrated on certain branches of a phylogenetic tree? Evolution Int. J. Org Evolution 44:539–557. doi: 10.1111/j.1558-5646.1990.tb05937.x 28567979

[CIT0079] Martínez-Noël G.M.A. , Cumino A.C., de los Angeles Kolman M., Salerno G.L. 2013. First evidence of sucrose biosynthesis by single cyanobacterial bimodular proteins. FEBS Lett. 587:1669–1674. doi: 10.1016/j.febslet.2013.04.012 23619081

[CIT0080] Math.NET Team. 2009. Math.NET Numerics. Available from: URL https://numerics.mathdotnet.com/

[CIT0081] McElreath R. 2020. Statistical rethinking. A Bayesian course with examples in R and STAN. Second ed. New York: Chapman and Hall/CRC. doi: 10.1201/9780429029608

[CIT0082] Mehltreter K. , Wachter H., Trabi C., Testo W., Sundue M., Jansen S. 2022. Hydathodes in ferns: their phylogenetic distribution, structure and function. Ann. Bot. 130:331–344. doi: 10.1093/AOB/MCAC076 35696156 PMC9486916

[CIT0083] Mira A. , Ochman H., Moran N.A. 2001. Deletional bias and the evolution of bacterial genomes. Trends Genet. 17:589–596. doi: 10.1016/S0168-9525(01)02447-7 11585665

[CIT0084] Nakov T. , Boyko J.D., Alverson A.J., Beaulieu J.M. 2017. Models with unequal transition rates favor marine origins of cyanobacteria and photosynthetic eukaryotes. Proc. Natl. Acad. Sci. U. S. A. 114:E10606–E10607. doi: 10.1073/pnas.1716692114 29208725 PMC5740659

[CIT0085] Navarro-González R. , McKay C.P., Mvondo D.N. 2001. A possible nitrogen crisis for Archaean life due to reduced nitrogen fixation by lightning. Nature 412:61–64. doi: 10.1038/35083537 11452304

[CIT0086] Newton M.A. , Raftery A.E. 1994. Approximate Bayesian inference with the weighted likelihood bootstrap. J. Roy. Stat. Soc. Ser B: Statist. Methodol. 56:3–26. doi: 10.1111/J.2517-6161.1994.TB01956.X

[CIT0087] Nguyen L.-T. , Schmidt H.A., von Haeseler A., Minh B.Q. 2015. IQ-TREE: a fast and effective stochastic algorithm for estimating maximum-likelihood phylogenies. Mol. Biol. Evol. 32:268–274. doi: 10.1093/molbev/msu300 25371430 PMC4271533

[CIT0088] Nielsen R. 2002. Mapping mutations on phylogenies. Syst. Biol. 51:729–739. doi: 10.1080/10635150290102393 12396587

[CIT0089] O’meara B.C. , Beaulieu J.M. 2021. Potential survival of some, but not all, diversification methods. EcoEvoRxiv (Preprint). doi: 10.32942/OSF.IO/W5NVD

[CIT0090] Oaks J.R. , Cobb K.A., Minin V.N., Leaché A.D. 2019. Marginal likelihoods in phylogenetics: a review of methods and applications. Syst. Biol. 68:681. doi: 10.1093/SYSBIO/SYZ003 30668834 PMC6701458

[CIT0091] Ochman H. , Lawrence J.G., Grolsman E.A. 2000. Lateral gene transfer and the nature of bacterial innovation. Nature 405:299–304. doi: 10.1038/35012500 10830951

[CIT0092] Oliver T. , Sánchez-Baracaldo P., Larkum A.W., Rutherford A.W., Cardona T. 2021. Time-resolved comparative molecular evolution of oxygenic photosynthesis. Biochim. Biophys. Acta, Bioenerg. 1862:148400. doi: 10.1016/J.BBABIO.2021.148400 33617856 PMC8047818

[CIT0093] Pagel M. 1994. Detecting correlated evolution on phylogenies: a general method for the comparative analysis of discrete characters. Proc. R. Soc. Lond. B Biol. Sci. 255:37–45. doi: 10.1098/rspb.1994.0006

[CIT0094] Paradis E. , Claude J., Strimmer K. 2004. APE: analyses of phylogenetics and evolution in R language. Bioinformatics 20:289–290. doi: 10.1093/bioinformatics/btg412 14734327

[CIT0095] Planavsky N.J. , Crowe S.A., Fakhraee M., Beaty B., Reinhard C.T., Mills B.J.W., Holstege C., Konhauser K.O. 2021. Evolution of the structure and impact of Earth’s biosphere. Nat. Rev. Earth Environ. 2:123–139. doi: 10.1038/s43017-020-00116-w

[CIT0096] Ponce-Toledo R.I. , Deschamps P., López-García P., Zivanovic Y., Benzerara K., Moreira D. 2017. An early-branching freshwater cyanobacterium at the origin of plastids. Curr. Biol. 27:386–391. doi: 10.1016/J.CUB.2016.11.056 28132810 PMC5650054

[CIT0097] Pufahl P.K. , Hiatt E.E. 2012. Oxygenation of the Earth’s atmosphere–ocean system: a review of physical and chemical sedimentologic responses. Mar. Pet. Geol. 32:1–20. doi: 10.1016/J.MARPETGEO.2011.12.002

[CIT0098] Rambaut A. , Drummond A.J., Xie D., Baele G., Suchard M.A. 2018. Posterior summarization in Bayesian phylogenetics using tracer 1.7. Syst. Biol. 67:901–904. doi: 10.1093/sysbio/syy032 29718447 PMC6101584

[CIT0099] Raven J.A. , Sánchez-Baracaldo P. 2021. *Gloeobacter* and the implications of a freshwater origin of cyanobacteria. Phycologia 60:402–418. doi: 10.1080/00318884.2021.1881729

[CIT0100] Reed R.H. , Borowitzka L.J., Mackay M.A., Chudek J.A., Foster R., Warr S.R.C., Moore D.J., Stewart W.D.P. 1986. Organic solute accumulation in osmotically stressed cyanobacteria. FEMS Microbiol. Rev. 2:51–56. doi: 10.1111/J.1574-6968.1986.TB01842.X

[CIT0101] Reed R.H. , Richardson D.L., Warr S.R.C., Stewart W.D.P. 1984. Carbohydrate accumulation and osmotic stress in cyanobacteria. Microbiology 130:1–4. doi: 10.1099/00221287-130-1-1

[CIT0102] Reeves J.C. , Wogelius R.A., Keating J.N., Sansom R.S. 2023. *Lasanius*, an exceptionally preserved Silurian jawless fish from Scotland. Palaeontology 66:e12643. doi: 10.1111/PALA.12643

[CIT0103] Ronquist F. , Huelsenbeck J.P. 2003. MrBayes 3: Bayesian phylogenetic inference under mixed models. Bioinformatics 19:1572–1574. doi: 10.1093/bioinformatics/btg180 12912839

[CIT0104] Royer-Carenzi M. , Pontarotti P., Didier G. 2013. Choosing the best ancestral character state reconstruction method. Math. Biosci. 242:95–109. doi: 10.1016/J.MBS.2012.12.003 23276531

[CIT0105] Sánchez-Baracaldo P. 2015. Origin of marine planktonic cyanobacteria. Sci. Rep. 5:17418. doi: 10.1038/srep17418 26621203 PMC4665016

[CIT0106] Sánchez-Baracaldo P. , Bianchini G., Di Cesare A., Callieri C., Chrismas N.A.M. 2019. Insights into the evolution of picocyanobacteria and phycoerythrin genes (mpeBA and cpeBA). Front. Microbiol. 10:45. doi: 10.3389/fmicb.2019.00045 30761097 PMC6363710

[CIT0107] Sánchez-Baracaldo P. , Bianchini G., Huelsenbeck J.P., Raven J.A., Pisani D., Knoll A.H. 2017a. Reply to Nakov *et al*.: Model choice requires biological insight when studying the ancestral habitat of photosynthetic eukaryotes. Proc. Natl. Acad. Sci. U.S.A. 114:E10608–E10609. doi: 10.1073/PNAS.1717417114 29208724 PMC5740660

[CIT0108] Sánchez-Baracaldo P. , Bianchini G., Wilson J.D., Knoll A.H. 2022. Cyanobacteria and biogeochemical cycles through Earth history. Trends Microbiol. 30:143–157. doi: 10.1016/J.TIM.2021.05.008 34229911

[CIT0109] Sánchez-Baracaldo P. , Cardona T. 2020. On the origin of oxygenic photosynthesis and cyanobacteria. New Phytol. 225:1440–1446. doi: 10.1111/nph.16249 31598981

[CIT0110] Sánchez-Baracaldo P. , Hayes P.K., Blank C.E. 2005. Morphological and habitat evolution in the cyanobacteria using a compartmentalization approach. Geobiology 3:145–165. doi: 10.1111/j.1472-4669.2005.00050.x

[CIT0111] Sánchez-Baracaldo P. , Raven J.A., Pisani D., Knoll A.H. 2017b. Early photosynthetic eukaryotes inhabited low-salinity habitats. Proc. Natl. Acad. Sci. U. S. A. 114:E7737–E7745. doi: 10.1073/pnas.1620089114 28808007 PMC5603991

[CIT0112] Sánchez-Baracaldo P. , Ridgwell A., Raven J.A. 2014. A Neoproterozoic transition in the marine nitrogen cycle. Curr. Biol. 24:652–657. doi: 10.1016/j.cub.2014.01.041 24583016

[CIT0113] Scanlan D.J. , Ostrowski M., Mazard S., Dufresne A., Garczarek L., Hess W.R., Post A.F., Hagemann M., Paulsen I., Partensky F. 2009. Ecological genomics of marine picocyanobacteria. Microbiol. Mole. Biol. Rev.: MMBR 73:249–299. doi: 10.1128/MMBR.00035-08 PMC2698417 19487728

[CIT0114] Schad M. , Konhauser K.O., Sánchez-Baracaldo P., Kappler A., Bryce C. 2019. How did the evolution of oxygenic photosynthesis influence the temporal and spatial development of the microbial iron cycle on ancient Earth? Free Radic. Biol. Med. 140:154–166. doi: 10.1016/J.FREERADBIOMED.2019.07.014 31323314

[CIT0115] Schirrmeister B.E. , de Vos J.M., Antonelli A., Bagheri H.C. 2013. Evolution of multicellularity coincided with increased diversification of cyanobacteria and the Great Oxidation Event. Proc. Natl. Acad. Sci. U. S. A. 110:1791–1796. doi: 10.1073/pnas.1209927110 23319632 PMC3562814

[CIT0116] Schirrmeister B.E. , Gugger M., Donoghue P.C.J. 2015. Cyanobacteria and the Great Oxidation Event: evidence from genes and fossils. Palaeontology 58:769–785. doi: 10.1111/pala.12178 26924853 PMC4755140

[CIT0117] Schirrmeister B.E. , Sanchez-Baracaldo P., Wacey D. 2016. Cyanobacterial evolution during the Precambrian. Int. J. Astrobiol. 15:187–204. doi: 10.1017/S1473550415000579

[CIT0118] Schopf J.W. 2013. The fossil record of cyanobacteria. In Whitton, B.A. editor. Ecology of Cyanobacteria II: their diversity in space and time. Dordrecht: Springer Netherlands. p. 15–36. doi: 10.1007/978-94-007-3855-3_2.

[CIT0119] Schultz T.R. , Churchill G.A. 1999. The role of subjectivity in reconstructing ancestral character states: a Bayesian approach to unknown rates, states, and transformation asymmetries. Syst. Biol. 48:651–664. doi: 10.1080/106351599260229

[CIT0120] Schwarz G. 1978. Estimating the dimension of a model. Ann. Stat. 6:461–464. doi: 10.1214/aos/1176344136

[CIT0121] Seo P.-S. , Yokota A. 2003. The phylogenetic relationships of cyanobacteria inferred from 16S rRNA, gyrB, rpoC1 and rpoD1 gene sequences. J. Gen. Appl. Microbiol. 49:191–203. doi: 10.2323/jgam.49.191 12949700

[CIT0122] Shih P.M. , Wu D., Latifi A., Axen S.D., Fewer D.P., Talla E., Calteau A., Cai F., Tandeau de Marsac N., Rippka R., Herdman M., Sivonen K., Coursin T., Laurent T., Goodwin L., Nolan M., Davenport K.W., Han C.S., Rubin E.M., Eisen J.A., Woyke T., Gugger M., Kerfeld C.A. 2013. Improving the coverage of the cyanobacterial phylum using diversity-driven genome sequencing. Proc. Natl. Acad. Sci. U. S. A. 110:1053–1058. doi: 10.1073/pnas.1217107110 23277585 PMC3549136

[CIT0123] Simão F.A. , Waterhouse R.M., Ioannidis P., Kriventseva E.V., Zdobnov E.M. 2015. BUSCO: assessing genome assembly and annotation completeness with single-copy orthologs. Bioinformatics 31:3210–3212. doi: 10.1093/bioinformatics/btv351 26059717

[CIT0124] Soo R.M. , Hemp J., Parks D.H., Fischer W.W., Hugenholtz P. 2017. On the origins of oxygenic photosynthesis and aerobic respiration in Cyanobacteria. Science (New York, N.Y.) 355:1436–1440. doi: 10.1126/science.aal3794 28360330

[CIT0125] Soo R.M. , Skennerton C.T., Sekiguchi Y., Imelfort M., Paech S.J., Dennis P.G., Steen J.A., Parks D.H., Tyson G.W., Hugenholtz P. 2014. An expanded genomic representation of the phylum Cyanobacteria. Genome Biol. Evol. 6:1031–1045. doi: 10.1093/gbe/evu073 24709563 PMC4040986

[CIT0126] Strom A.R. , Kaasen I. 1993. Trehalose metabolism in *Escherichia coli*: stress protection and stress regulation of gene expression. Mol. Microbiol. 8:205–210. doi: 10.1111/j.1365-2958.1993.tb01564.x 8391102

[CIT0127] Strunecký O. , Ivanova A.P., Mareš J. 2023. An updated classification of cyanobacterial orders and families based on phylogenomic and polyphasic analysis. J. Phycol. 59:12–51. doi: 10.1111/JPY.13304 36443823

[CIT0128] Stüeken E.E. , Kipp M.A., Koehler M.C., Buick R. 2016. The evolution of Earth’s biogeochemical nitrogen cycle. Earth-Sci. Rev. 160:220–239. doi: 10.1016/J.EARSCIREV.2016.07.007

[CIT0129] Sukenik A. , Zohary T., Padisák J. 2009. Cyanoprokaryota and other prokaryotic algae. Encyclopedia Inland Waters 1:138–148. doi: 10.1016/B978-012370626-3.00133-2

[CIT0130] Swanner E.D. , Mloszewska A.M., Cirpka O.A., Schoenberg R., Konhauser K.O., Kappler A. 2015. Modulation of oxygen production in Archaean oceans by episodes of Fe(II) toxicity. Nat. Geosci. 8:126–130. doi: 10.1038/ngeo2327

[CIT0131] Tautz D. , Domazet-Lošo T. 2011. The evolutionary origin of orphan genes. Nat. Rev. Genet. 12:692–702. doi: 10.1038/nrg3053 21878963

[CIT0132] Turchyn A.V. , Depaolo D.J. 2019. Seawater chemistry through phanerozoic time. Annu. Rev. Earth Planet. Sci. 47:197–224. doi: 10.1146/ANNUREV-EARTH-082517-010305

[CIT0133] Uyeda J.C. , Harmon L.J., Blank C.E. 2016. A comprehensive study of cyanobacterial morphological and ecological evolutionary dynamics through deep geologic time. PLoS One 11:e0162539. doi: 10.1371/JOURNAL.PONE.0162539 27649395 PMC5029880

[CIT0134] Wagenmakers E.J. , Farrell S. 2004. AIC model selection using Akaike weights. Psychon. Bull. Rev. 11:192–196. doi: 10.3758/BF03206482/METRICS 15117008

[CIT0135] Wasserman L. 2000. Bayesian model selection and model averaging. J. Math. Psychol. 44:92–107. doi: 10.1006/JMPS.1999.1278 10733859

[CIT0136] Xie W. , Lewis P.O., Fan Y., Kuo L., Chen M.-H. 2011. Improving marginal likelihood estimation for Bayesian phylogenetic model selection. Syst. Biol. 60:150–160. doi: 10.1093/sysbio/syq085 21187451 PMC3038348

[CIT0137] Zhang T. , Rurik I., Vďačný P. 2023. A holistic approach to inventory the diversity of mobilid ciliates (Protista: Ciliophora: Peritrichia). Org. Divers. Evol. 23:425–454. doi: 10.1007/S13127-022-00601-8

